# Direct antiglobulin test for the prediction of neonatal hyperbilirubinemia needing an intervention: a systematic review and diagnostic test accuracy meta-analysis

**DOI:** 10.3389/fped.2024.1475623

**Published:** 2025-01-28

**Authors:** Vijay Kumar Krishnegowda, Viraraghavan Vadakkencherry Ramaswamy, Thangaraj Abiramalatha, Tapas Bandyopadhyay, Abdul Kareem Pullattayil S, Prakash Kannan Loganathan

**Affiliations:** ^1^ Department of Neonatology, Institute of Medical Sciences and SUM Hospital, Bhubaneswar, Orissa, India; ^2^ Department of Neonatology, Ankura Hospital for Women and Children, Hyderabad, Telangana, India; ^3^ Department of Neonatology, Kovai Medical Center and Hospital (KMCH), Coimbatore, Tamil Nadu, India; ^4^ KMCH Research Foundation, Coimbatore, Tamil Nadu, India; ^5^ Department of Neonatology, ABVIMS and Dr. RML Hospital, New Delhi, India; ^6^ Health Sciences Librarian, Queen’s University, Kingston, ON, Canada; ^7^ Neonatal Unit, James Cook University Hospital, Middlesbrough, United Kingdom; ^8^ Clinical Academic Office, Faculty of Medical Sciences, Newcastle University, Newcastle, United Kingdom; ^9^ Department of Physics, University of Durham, Durham, United Kingdom

**Keywords:** neonatal jaundice, Coombs test, meta-analysis, newborn, exchange transfusion

## Abstract

**Importance:**

The direct antiglobulin test (DAT) is commonly used as a screening test for predicting significant neonatal hyperbilirubinemia requiring intervention. However, evidence for this approach is limited.

**Objective:**

The aim of this study was to evaluate the diagnostic utility of DAT in predicting the need for phototherapy and double volume exchange transfusion (DVET) in neonates with ABO and Rhesus (Rh) incompatibility conditions.

**Methods:**

MEDLINE, Embase, CENTRAL, CINAHL, and Web of Science were searched from inception until 1 February 2024. Randomized controlled trials (RCTs) and non-RCTs were eligible for inclusion. Two reviewers screened the titles and abstracts blinded to each other. A Bayesian bivariate random-effects model was employed for the diagnostic test accuracy meta-analyses. Risk of bias was assessed using Quality Assessment for Studies of Diagnostic Accuracy 2 and certainty of evidence (CoE) was adjudged according to the Grading of Recommendations, Assessment, Development, and Evaluations (GRADE) guidelines.

**Results:**

In total, 53 studies were included in the systematic review and 28 were synthesized in the meta-analysis. For the need for phototherapy outcome, the pooled sensitivity [95% credible interval (CrI)] and specificity (95% CrI) of DAT in ABO incompatibility (18 studies, *n* = 10,110) were 56.1% (44.5%–67.8%) and 83.6% (71.6%–90.8%). For Rh incompatibility (three studies, *n* = 491), the sensitivity and specificity were 40.4% (12.2%–81.7%) and 89.9% (72.7%–94.6%). The CoE was predominantly low. For the need for DVET outcome, the pooled sensitivity and specificity of DAT in ABO incompatibility (three studies, *n* = 2,652) were 83.6% (35.8%–99.6%) and 74.5% (40.3%–92.7%). For Rh incompatibility (two studies, *n* = 240), the sensitivity and specificity were 80.3% (34.2%–97.3%) and 68.0% (25.3%–92.1%). The CoE was predominantly very low.

**Conclusion:**

In ABO and Rh incompatibility, DAT probably has moderate specificity and low sensitivity for predicting the need for phototherapy. For DVET, though DAT is possibly a better predictor due to its acceptable sensitivity, the predictive interval was wide. Thus, we do not suggest the routine use of DAT screening to predict the need for phototherapy and DVET. However, it may be used as a second-tier investigation for risk stratification of high-risk neonates.

**Systematic Review Registration:**

https://www.crd.york.ac.uk/prospero/display_record.php?ID=CRD42022297785, PROSPERO (CRD42022297785).

## 1 Introduction

Neonatal hyperbilirubinemia (NNH) is a common diagnosis in neonates, requiring treatment in the initial postnatal days (1). While the majority of these neonates needing treatment are managed with phototherapy, a small proportion may require additional therapies such as intravenous immunoglobulin (IVIG) and double volume exchange transfusion (DVET). Despite the availability of effective interventions, the risk of serious adverse outcomes including kernicterus, cerebral palsy, hearing loss, and even mortality remains a significant concern (2). These adverse outcomes could be prevented by the timely identification and treatment of neonates at risk of developing severe NNH.

Evidence-based guidelines have recommended the use of various screening tools for early identification and treatment of NNH (1, 3). Despite the incorporation of these tools into clinical practice, instances of serious adverse outcomes secondary to NNH have been reported. This is often attributed to factors such as challenges in identifying high-risk neonates, limitations of the screening tools, and adaptation of policies such as early discharge from healthcare facilities. As a result, regional guidelines suggest risk stratification based on gestational age, bilirubin levels, and the presence of various other morbidities such as sepsis, asphyxia, and ABO, Rhesus (Rh), and other blood group incompatibilities (1, 4, 5). The inclusion of additional tests such as the direct antiglobulin test (DAT) has also been recommended (1, 5).

DAT is commonly performed in suspected cases of hemolytic disease of the newborn due to ABO or Rh incompatibility (6). However, the current clinical guideline does not recommend routine testing of umbilical cord blood for DAT in ABO and Rh incompatibility (5). As the evidence for the same is contentious, many centers continue to use routine DAT testing as a screening test to identify at-risk neonates (6–10).

Thus, we conducted a systematic review and diagnostic test accuracy (DTA) meta-analyses with an aim to evaluate the diagnostic utility of DAT in predicting NNH requiring treatment in neonates with ABO and Rh incompatibility conditions.

## 2 Methods

The protocol was registered with PROSPERO (CRD42022297785) (11), and the reporting is in accordance with the Preferred Reporting Items for Systematic Reviews and Meta-Analyses of Diagnostic Test Accuracy Studies (PRISMA-DTA) (12).

### 2.1 Study eligibility

Randomized controlled trials (RCTs), non-RCTs, and conference abstracts were eligible for inclusion. Studies that evaluated the diagnostic performance of DAT in predicting the need for treatment of NNH in various mother-neonate blood group pairs, including but not exclusively ABO and Rh incompatibility, were also eligible for inclusion. Case reports, traditional reviews, and systematic reviews were excluded. There were no language restrictions.

### 2.2 Patient population

We included studies conducted in different healthcare settings in late preterm and term neonates. Studies reporting on preterm neonates of <34 weeks' gestation, NNH secondary to enzyme deficiency, sepsis, liver disorders, and those who had received fetal therapy were excluded.

### 2.3 Index test

A DAT was performed on umbilical cord blood or during the first 14 days of postnatal life. We included studies that had used the two commonly used methods of DAT measurement (gel or tube agglutination methods). For the generation of the 2 × 2 table, we considered a weakly positive DAT as a positive test.

### 2.4 Reference standard

A serum bilirubin test was performed in the first 14 days of postnatal life.

### 2.5 Target condition

We considered the following outcomes: the need for phototherapy, DVET, and IVIG therapy in the first 14 days, irrespective of the treatment thresholds recommended by different guidelines. We did not include outcome parameters such as clinical jaundice and significant hyperbilirubinemia not requiring intervention.

### 2.6 Information sources

We conducted a comprehensive search of MEDLINE, Embase, CENTRAL, CINAHL, and Web of Science from inception till 1 February 2024. (AS, VK) (Supplementary Table 1). In addition, we searched the bibliographies of included studies and review articles to identify potentially eligible studies for inclusion.

### 2.7 Study selection and data extraction

All the study titles and abstracts were screened independently in duplicates by two authors (TA, TB) using an online software platform (Rayyan QCRI, Doha) (13). Data extraction was independently performed by the two investigators (VR, VK) using a preformed data extraction form, and disagreements were resolved by consensus. We recorded data items, including blood group, timing of DAT measurement, outcomes, and test accuracy measures [true positives (TPs), true negatives (TNs), false positives (FPs), and false negatives (FNs)].

### 2.8 Study quality assessment

The risk of bias and applicability concerns of the included studies were assessed using the Quality Assessment for Studies of Diagnostic Accuracy 2 tool (QUADAS-2) (14). Two authors (VK, PK) independently assessed the quality of the studies.

### 2.9 Data synthesis

Data synthesis was conducted in accordance with the Cochrane Handbook for Systematic Reviews of Diagnostic Test Accuracy (15). We estimated individual sensitivities and specificities, along with their 95% confidence intervals using DTA measures. These findings are presented using forest plots and the summary receiver operating characteristic (SROC) space. In addition, we used a Bayesian bivariate random-effects approach to estimate median pooled sensitivity and specificity, along with 95% credible intervals (CrI). Bayesian analysis was performed using METABayesDTA version 1.4 and R software version 4.3.1 (R Foundation for Statistical Computing, Vienna, Austria) using the “meta4diag' and “meta” packages (16, 17). Vague priors were used. A Stan sampler with two chains, 500 warm-up iterations, and 1,500 total iterations was utilized. Model fit was assessed by visualizing the correlation residual plot and the frequency table probability residual plot. Model convergence was confirmed by examining the trace and density plots.

A meta-analysis was performed for each outcome: the need for phototherapy, the need for DVET, and the requirement of IVIG therapy. Within each outcome, the DTA measures were estimated for specific maternal and neonatal blood group combinations. For the DTA meta-analysis, we included only studies with ABO or Rh incompatibility, as these are the two commonly encountered clinical scenarios. Studies that evaluated DAT in blood group scenarios other than ABO or Rh incompatibility were included in the narrative review. ABO or Rh blood group incompatibilities were categorized into ABO incompatibility [Mother blood group (MBG): O; Rh positive and baby blood group (BBG): A or B irrespective of Rh status], Rh incompatibility (MBG: A, or B or AB or O with Rh negative and BBG: A or B or AB or O with Rh positive), or ABO/Rh incompatibility (MBG: O with Rh positive and BBG: A or B with Rh positive or negative OR MBG: A, B, AB, O with Rh negative and BBG: A or B or AB or O with Rh positive).

#### 2.9.1 Subgroup and sensitivity analysis

Heterogeneity was explored using subgroup analysis of the covariates, DAT testing methods and treatment thresholds, as per the guidelines. We performed a *post-hoc* sensitivity analysis by excluding studies with applicability concerns in the patient selection domain. For publication bias, we examined the regression coefficient line for plot asymmetry. A funnel plot of the log diagnostic odds ratio against 1/(effective sample size)^1/2^ was generated. For assessing the certainty of the evidence, we used GRADEPro, following GRADE guidelines for DTA meta-analysis for each target condition and blood group combination (18).

## 3 Results

### 3.1 Study selection

**Figure 1:**
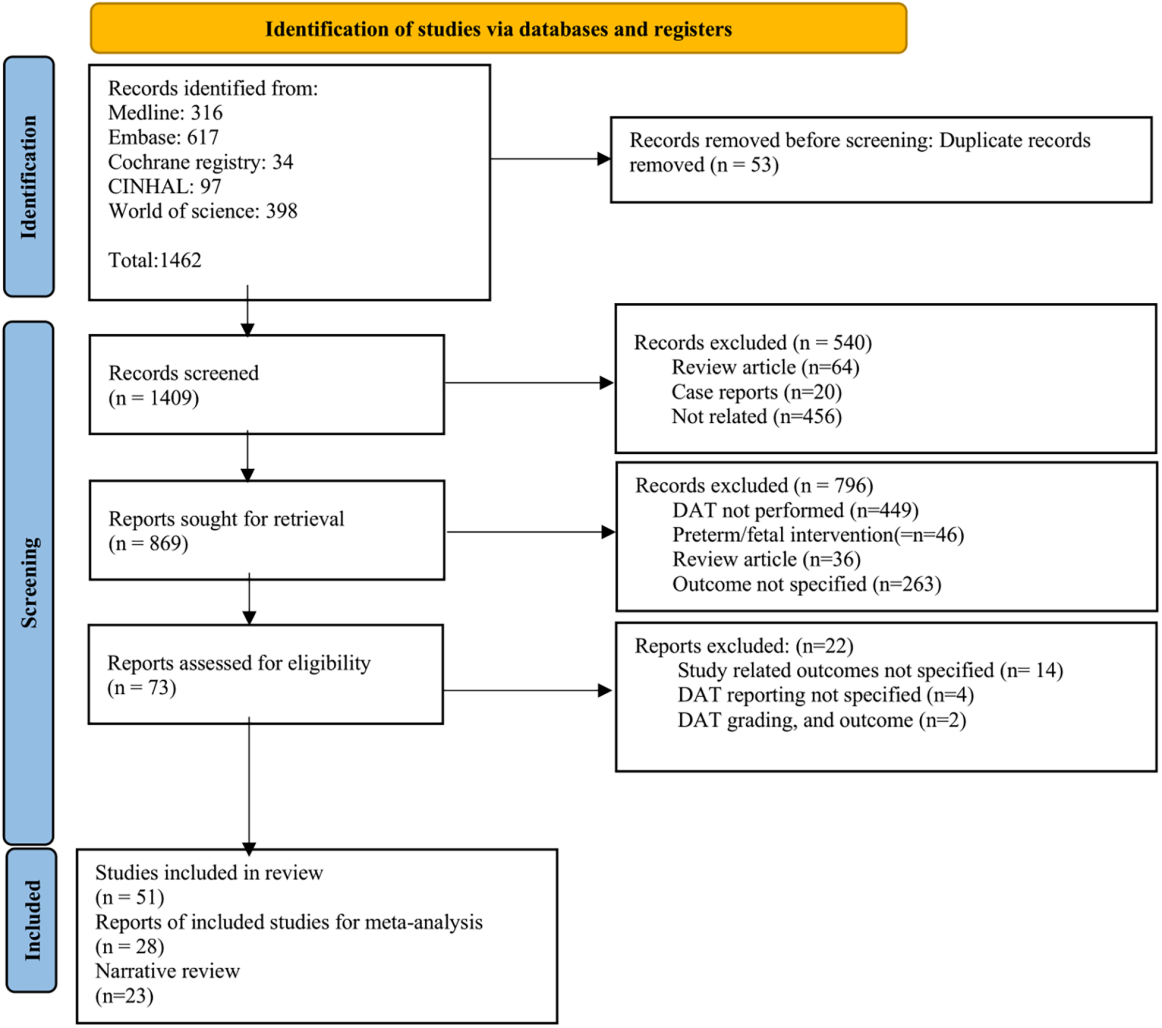
PRISMA flow chart of the literature search.

After the removal of duplicates, 1,409 titles and abstracts were screened. Of these, 73 were selected for full-text review. Among them, 51 were included in the systemic review. Of the included studies, 28 were synthesized in a DTA meta-analysis, and 23 studies were included in the narrative review (Figure 1). The excluded studies with valid reasons for their exclusion are listed in Supplementary Table 2.

### 3.2 Characteristics of included studies

A total of 28 studies (*n* = 20,935) (10, 19–44) were included in the meta-analysis, with 20 studies (10, 19, 26, 28, 30, 32–44) (*n* = 11,385), 3 studies (10, 22, 29) (*n* = 491), and 9 studies (10, 20, 21, 23–25, 27, 31) (*n* = 9,561) assessed DAT in the ABO, Rh and ABO/Rh incompatibility scenarios, respectively. Only two studies (23, 25) included late preterm along with term neonates, while the others only included term neonates. Furthermore, 16 studies (20, 22, 24, 25, 28–33, 36, 39–42, 45) did not specify the method of DAT assessment, 7 studies (10, 19, 23, 27, 35, 37, 38) utilized the gel method, and 5 studies (21, 26, 34, 43, 44) used the column agglutination method. Most DAT assessments were performed with cord blood samples (10, 19, 20, 22–31, 33–37, 40–44), while two studies (21, 38) performed the assessments during the initial postnatal days. Of the included studies, 28 studies (10, 19–44) (*n* = 20,162) reported the outcome measure of the need for phototherapy, 7 studies (19, 22, 27, 29, 31, 32, 44) (*n* = 3,338) were on DVET, and 2 studies (19, 45) (*n* = 1,005) were on IVIG therapy. The characteristics of the included studies synthesized in the meta-analysis are provided in Table 1, and those in the narrative review are summarized in Appendix I in the Supplementary Material.

**Table 1:** Characteristics of included studies in the meta-analysis.

No	Author, study design, country	Sample size	Gestation (mean/median)	Blood group scenario	Index test (DAT method and timing)	Reference standard (Bilirubin/Tcb/clinical examination and timing)	Target condition (Treatment threshold)
1	Secil, 2024 Retrospective Turkey	820	38.8 (1.3) wks	ABO^a^	Method: gel, Timing: cord	Tcb followed by Bilirubin till 96 h	Phototherapy, Threshold: AAP
2	Daunov, 2024 Retrospective United States	579	39 (38–40) wks	ABO^a^	Method: NS, Timing: cord	Bilirubin	IVIG, Threshold: AAP
3	Novoselac, 2023 Retrospective Croatia	182	39 (39–40) wks	ABO/Rh negative^c^	Method: column agglutination, Timing: cord (Rh negative) and clinical jaundice (ABO)	Bilirubin (clinical jaundice)	Phototherapy, Threshold: AAP
4	Omran, 2023 Retrospective Saudi Arabia	611	39 wks	ABO^a^	Method: NS, Timing: cord	Tcb followed by Bilirubin	Phototherapy, Threshold: AAP
5	Gabbay, 2023 Retrospective United States	542	39.4 (36–41.7) wks	ABO/Rh negative^c^	Method: NS, Timing: cord	Bilirubin or Tcb at 12 and 24 h	Phototherapy, Threshold: AAP
6	Duete, 2022 Retrospective Brazil	8	NS	Rh negative^b^	Method: NS, Timing; cord	Bilirubin	Phototherapy/DVET, Threshold: NS
7	Chowdhary, 2022 Retrospective India	426	38.4 (37.5–39.2) wks	ABO^a^	Method: gel, Timing: cord	Bilirubin at 24 h (DAT positive) and clinical jaundice	Phototherapy/DVET/IVIG, Threshold: AAP
8	Kardum, 2020 Retrospective Croatia	1360	39 (38–40) wks	ABO/Rh negative^c^	Method: NS, Timing: cord	Bilirubin or Tcb within 2 days	Phototherapy, Threshold: NICE
9	Alkhater, 2021 Retrospective Saudi Arabia	251	38 (1.4) wks	ABO/Rh negative	Method: gel, Timing: cord	Bilirubin [Clinical jaundice, positive DAT, hospital protocol (IDM and SGA)]	Phototherapy, Threshold: NICE
10	Mehta, 2021 Retrospective United States	158	Late preterm and term	ABO/Rh negative^c^	Method: gel, Timing: cord	Bilirubin before discharge	Phototherapy, Threshold: AAP
11	Fei, 2020 Retrospective United States	100	39.1 (1.4) wks	ABO^a^	Method: column agglutination (Fei_A); gel (Fei_B) Timing: cord	Bilirubin	Phototherapy, Threshold: AAP
12	Ligsay, 2020 Retrospective United States	6,622	Late preterm and term	ABO/Rh negative^c^	Method: NS, Timing: cord	Bilirubin	Phototherapy, Threshold: AAP
13	Shin, 2019 Retrospective South Korea	303	38 wks	ABO/Rh negative^c^	Method: gel, Timing: cord	Bilirubin	Phototherapy/DVET, Threshold: AAP
14	Altuntas, 2019 Prospective Turkey	83	38.9 (1.2) wks	ABO^a^	Method: NS, Timing: cord	Bilirubin at 6, 24 h, and until 15 days	Phototherapy, Threshold: AAP
15	Zonneveld, 2017 Prospective Suriname	232	38 wks	Rh negative^b^	Method: NS, Timing: cord	Bilirubin	Phototherapy, Threshold: NICE
16	Schutzman, 2010 Retrospective	700	39 (0.48) wks	ABO^a^	Method: NS, Timing: cord	Bilirubin (cord sample followed every 8 h in positive DAT)	Phototherapy, Threshold: AAP
17	Tatopoulos 2010 Retrospective United States	257	Term	ABO^a^	Method: gel, Timing: day 1	Bilirubin	Phototherapy/DVET, Threshold: AAP
18	Bakkeheim, 2009 Prospective Norway	98	39.8 (39.3–40.3) wks	ABO^a^	Method: gel, Timing: cord	Bilirubin (clinical jaundice)	Phototherapy/DVET/IVIG, Threshold: Norwegian
19	Sarici, 2002 Prospective Turkey	136	39.2 (1.1) wks	ABO^a^	Method: NS, Timing: NS	Bilirubin	Phototherapy, Threshold: AAP
20	Geelkerken, 1999 Retrospective Netherlands	143	NS	ABO/Rh negative^c^	Method: NS, Timing: cord	Bilirubin	Phototherapy/DVET, Threshold: NS
21	Dudin, 1993 Retrospective Palestinian	1,530	38 wks	ABO^a^	Method: NS, Timing: NS	Bilirubin within the first week	Phototherapy/DVET, Threshold: NS
22	Diane, 1993 Retrospective United States	143	39 (1.3) wks	ABO^a^	Method: NS, Timing: cord	Bilirubin	Phototherapy, Threshold: Cockington
23	Comos, 1991 Retrospective Spain	1,933	37 wks	ABO^a^	Method: NS, Timing: cord	Bilirubin in first 2 days	Phototherapy, Threshold: Cockington
24	Brouwers, 1988 Prospective Netherlands	200	40 wks	ABO^a^	Method: NS, Timing: cord	Bilirubin	Phototherapy, Threshold: NS
25	Meberg, 1998 Prospective Norway	2,335	Term	ABO^a^	Method: NS, Timing: cord	Tcb followed by Bilirubin	Phototherapy, Threshold: Hillingdon hospital bilirubin chart
26	Han, 1988 Prospective Singapore	251	NS	ABO^a^	Method: column agglutination, Timing: cord	Bilirubin at 24 and 48 h	Phototherapy, Threshold: NS
27	Whyte, 1981 Prospective Scotland	142	Term	ABO^a^	Method: column agglutination, Timing: cord	Bilirubin	Phototherapy, Threshold: NS
28	Peevy, 1978 Retrospective United States	696	NS	ABO^a^	Method: column agglutination, Timing: cord	Bilirubin	DVET, Threshold: NS

Wks, weeks; DAT, direct antiglobin test; TcB, transcutaneous bilirubin; AAP, American Academy of Pediatrics; NS, not specified; IVIG, intravenous immunoglobulin; DVET, double volume exchange transfusion; Rh, Rhesus; NICE, National Institute for Health and Care Excellence; IDM, infant of diabetic mother; SGA, small for gestational age.

^a^ ABO: Mother blood group—O and Rhesus positive; and neonate blood group—A or B and Rhesus positive or negative.

^b^ Rhesus negative: Mother blood group—A, B, AB, or O and Rhesus negative; and neonate blood group—A, B, AB, or O and Rhesus positive.

^c^ ABO/Rhesus negative: Mother blood group—O and Rhesus positive; and neonate blood group—A or B and Rhesus positive or negative **OR** Mother blood group—A, B, AB, or O and Rhesus negative; and neonate blood group—A, B, AB, or O and Rhesus positive.

### 3.3 Risk of bias and applicability

**Figure 2:**
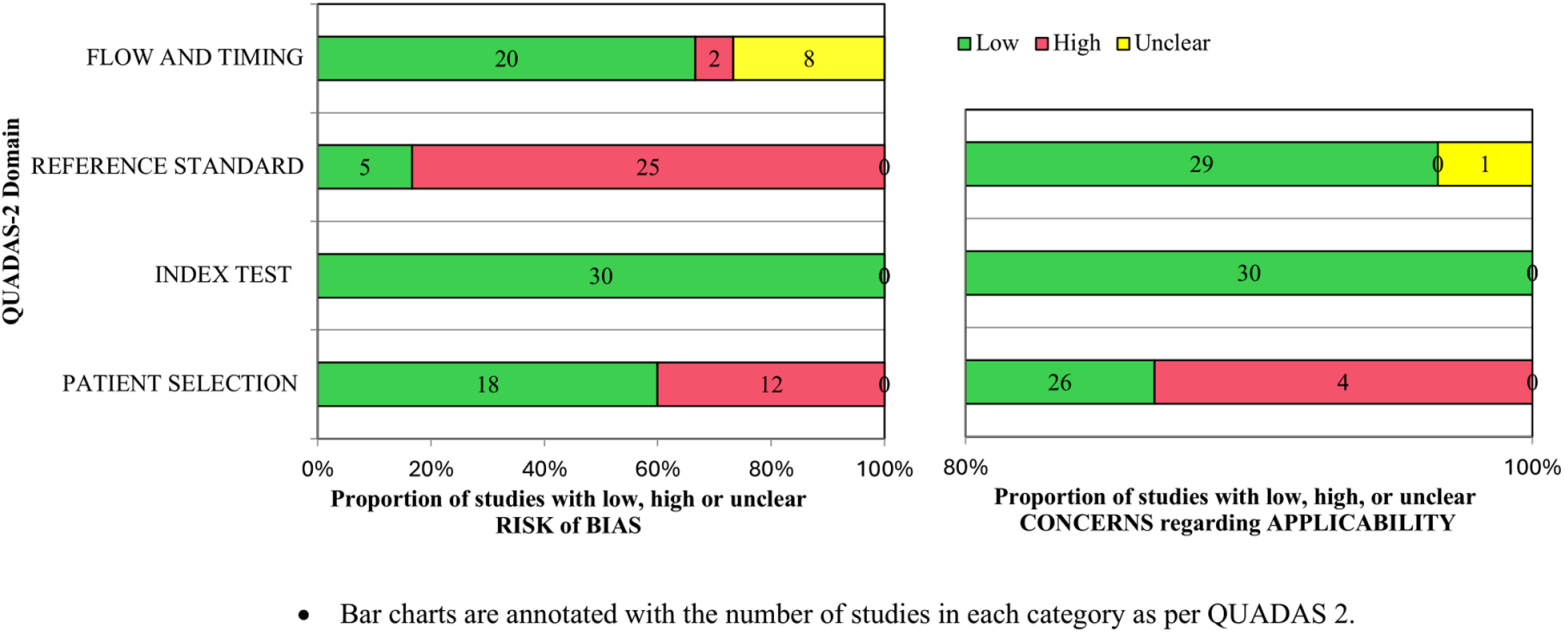
Proportions of studies with low, high, or unclear risk of bias and applicability concerns as per QUADAS-2.

Overall, the studies were adjudged to have a moderate risk of bias (Table 2). The common reason to adjudge studies as having a high risk of bias was in the domain of reference standard, as the DAT result was not blinded to the clinicians. For patient selection, high risk was attributed to the non-consecutive inclusion of neonates. Applicability concerns in the patient selection domain were related to the selective inclusion of neonates by prior testing. The proportion of studies assessed to have an overall risk of bias of “low,” “high,” or “unclear” is presented in Figure 2.

**Table 2:** QUADAS-2 assessment of the studies for risk of bias and applicability concerns.

		Risk of Bias				Applicability concerns		
	Study	rob_PS	rob_IT	rob_RS	rob_FT	ac_PS	ac_IT	ac_RS
1	Secil, 2024	High	Low	High	Low	Low	Low	Low
2	Daunov, 2024	Low	Low	High	Low	Low	Low	Low
3	Gabbay, 2023	Low	Low	High	Low	Low	Low	Low
4	Gabbay, 2023	Low	Low	High	Low	Low	Low	Low
5	Novoselac, 2023	High	Low	High	Unclear	High	Low	Low
6	Omran, 2023	High	Low	High	High	Low	Low	Low
7	Chowdhary, 2022	Low	Low	High	Low	Low	Low	Low
8	Duete, 2022	High	Low	High	Unclear	High	Low	Low
9	Alkhater, 2021	Low	Low	High	Unclear	Low	Low	Low
10	Mehta, 2021	Low	Low	High	Low	Low	Low	Low
11	Kardum, 2021	Low	Low	Low	Low	Low	Low	Low
12	Ligsay, 2020	Low	Low	High	Low	Low	Low	Low
13	Fei, 2020	High	Low	High	Low	Low	Low	Low
14	Fei, 2020	High	Low	High	Low	Low	Low	Low
15	Shin, 2019	High	Low	High	Unclear	High	Low	Low
16	Altuntas, 2019	Low	Low	High	Unclear	Low	Low	Low
17	Zonneveld, 2017	High	Low	Low	Low	Low	Low	Low
18	Schutzman, 2010	Low	Low	High	Unclear	Low	Low	Low
19	Tatopoulos, 2010	Low	Low	High	Low	Low	Low	Low
20	Bakkeheim, 2009	Low	Low	High	Low	High	Low	Low
21	Sarici, 2002	Low	Low	High	Low	Low	Low	Low
22	Geelkerken, 1999	High	Low	Low	High	Low	Low	Low
23	Meberg, 1998	Low	Low	High	Low	Low	Low	Low
24	Dudin, 1993	Low	Low	High	Unclear	Low	Low	Unclear
25	Diane, 1993	Low	Low	High	Low	Low	Low	Low
26	Comos, 1991	Low	Low	Low	Low	Low	Low	Low
27	Han, 1988	Low	Low	High	Low	Low	Low	Low
28	Brouwers, 1988	High	Low	High	Low	Low	Low	Low
29	Whyte, 1981	High	Low	High	Low	Low	Low	Low
30	Peevy, 1978	High	Low	Low	Unclear	Low	Low	Low

Rob, risk of bias; PS, patient selection; IT, index test; RS, reference standard; FT, flow and timing; ac, applicability concern.

### 3.4 Main results

We have summarized the study-specific sensitivity and specificity of all the included studies for the target conditions: the need for phototherapy (Supplementary Figures 1, 2), DVET (Supplementary Figures 3, 4), and IVIG therapy (Supplementary Figures 5, 6), employing forest plots and the SROC curves.

**Figure 3:**
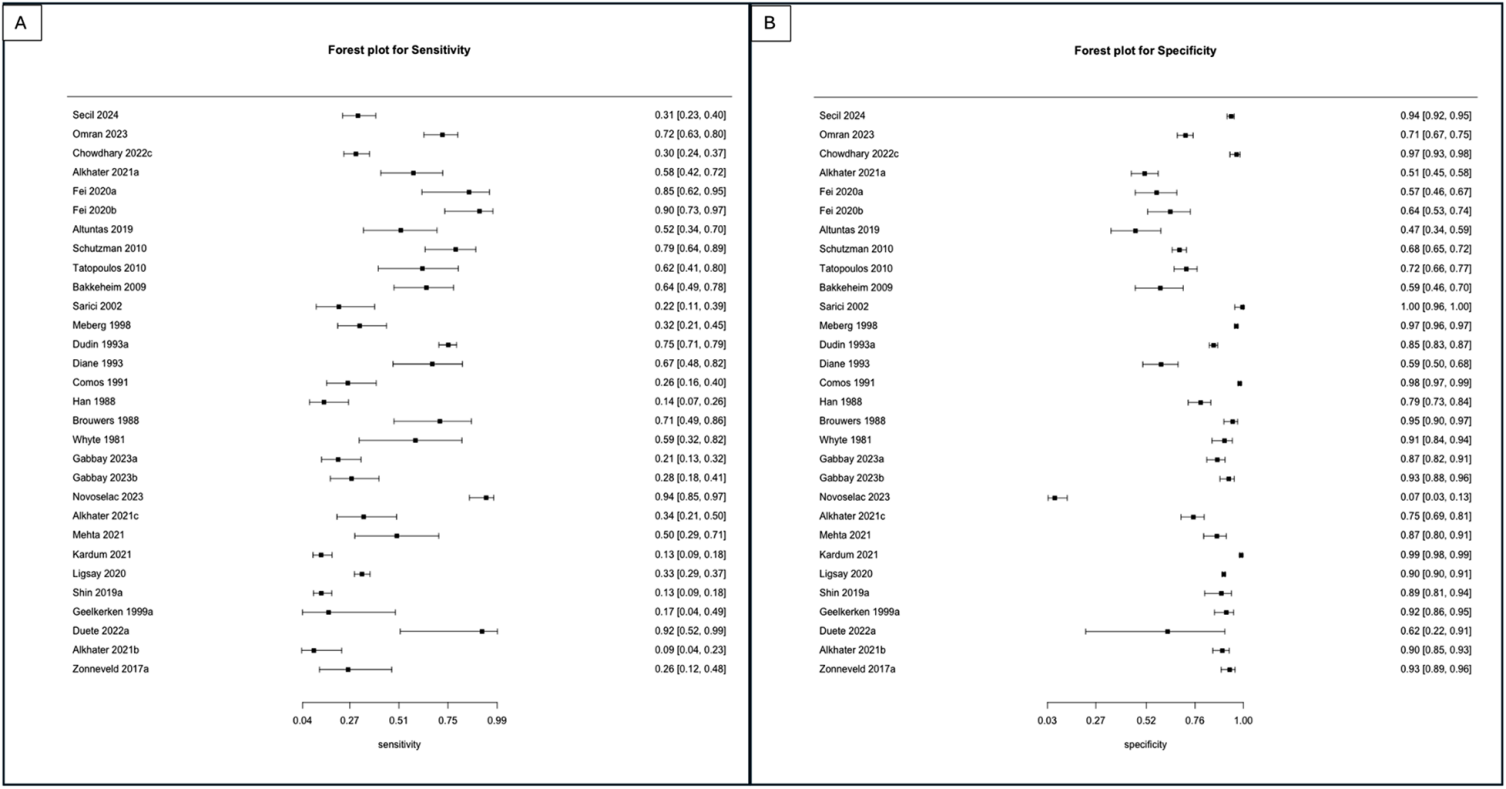
**(A)** Forest plot depicting the sensitivity of studies utilizing the DAT as an index test to predict the need for phototherapy in ABO, Rh, and ABO/Rh incompatibility scenarios. **(B)** Forest plot depicting the specificity of studies utilizing the DAT as an index test to predict the need for phototherapy in ABO, Rh, and ABO/Rh incompatibility scenarios.

**Figure 4:**
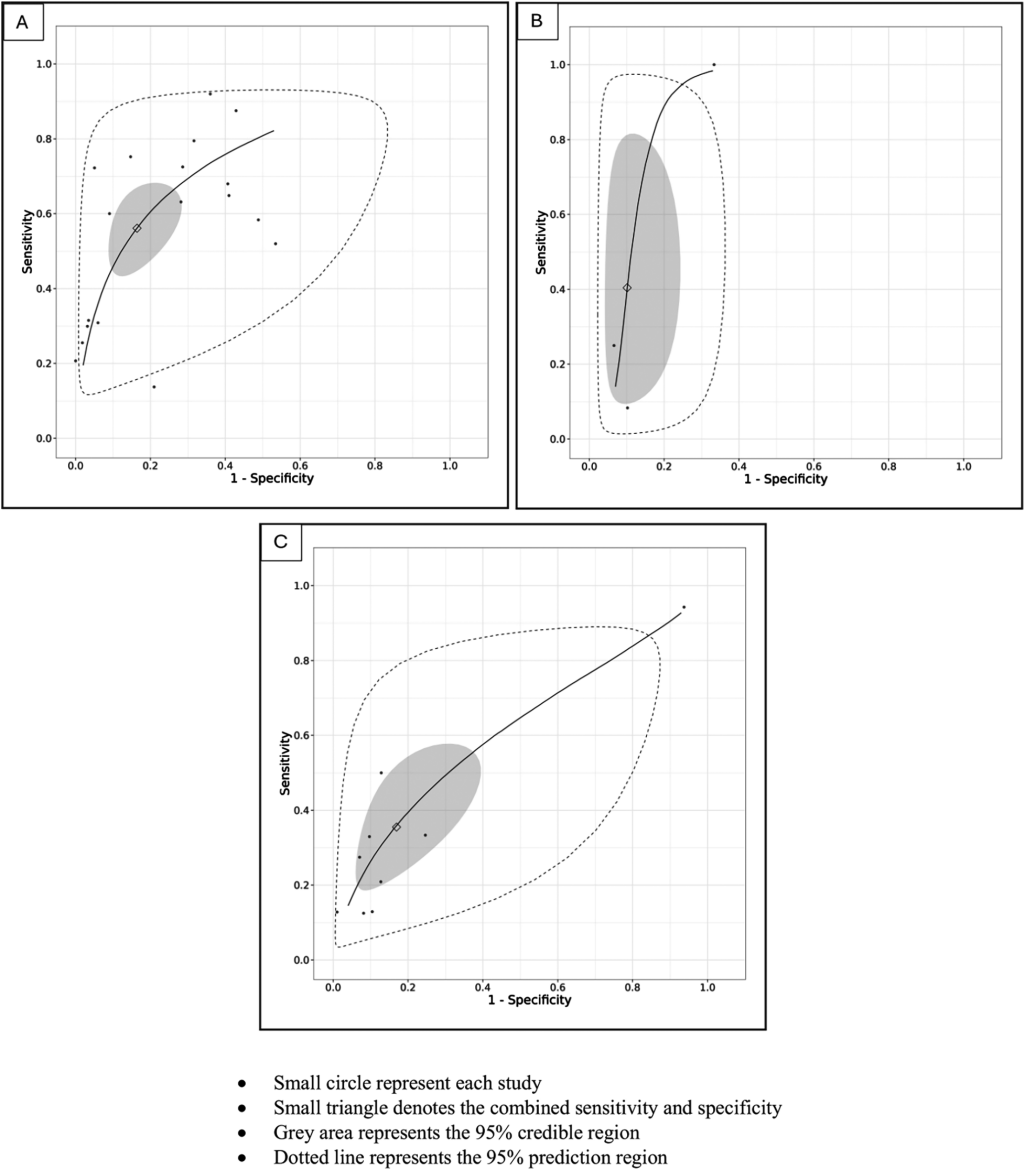
**(A)** SROC plot of studies utilizing the DAT as an index test to predict the need for phototherapy in ABO incompatibility scenarios. **(B)** SROC plot of studies utilizing the DAT as an index test to predict the need for phototherapy in Rh incompatibility scenarios. **(C)** SROC plot of studies utilizing the DAT as an index test to predict the need for phototherapy in ABO/Rh incompatibility scenarios.

For the primary target condition, the need for phototherapy, a total of 28 studies (*n* = 20,162) (10, 19–44) were included. In the meta-analysis of studies evaluating DAT in ABO incompatibility (18 studies; *n* = 10,110) (10, 19, 26, 28, 30, 32–43), the posterior median sensitivity and specificity were 56.1% (95% CrI: 44.5%–67.8%) and 83.6% (95% CrI: 71.6%–90.8%), respectively. For the Rh isoimmunization scenario (three studies; *n* = 491) (10, 22, 29), the pooled sensitivity and specificity were 40.4% (95% CrI: 12.2%–81.7%) and 89.9% (95% CrI: 72.7%–94.6%), respectively. Studies that had evaluated DAT in ABO/Rh incompatibility (nine studies; *n* = 9,561) (10, 20, 21, 23–25, 27, 31) yielded a pooled sensitivity and specificity of 35.8% (95% CrI: 19.6%–57.0%) and 82.5% (95% CrI: 61.9%–93.1%), respectively (Figures 3, 4, Table 3).

**Table 3:** Main analysis of studies based on target condition and blood group settings.

	Studies/participants	Blood group scenario	Sensitivity (95% CrI)	Specificity (95% CrI)	LR+ (95% CrI)	LR− (95% CrI)	DOR (95% CrI)
Phototherapy							
1	18 studies; *n* = 10,110	ABO incompatibility^a^	0.561 (0.445–0.678)	0.836 (0.716–0.908)	3.40 (2.08–5.72)	0.526 (0.399–0.668)	6.513 (3.36–12.20)
2	Three studies; *n* = 491	Rh incompatibility^b^	0.404 (0.122–0.817)	0.899 (0.727–0.946)	3.60 (0.919–10.56)	0.67 (0.207–1.013)	5.61 (0.90–42.32)
3	Nine studies; *n* = 9,561	ABO/Rh incompatibility^c^	0.358 (0.196–0.570)	0.825 (0.619–0.931)	2.055 (1.08–4.0)	0.782 (0.591–0.964)	2.687 (1.129–5.81)
Exchange transfusion							
1	Three studies; *n* = 2,652	ABO incompatibility^a^	0.836 (0.358–0.996)	0.745 (0.403–0.927)	2.917 (1.42–5.89)	0.290 (0.08–0.76)	10.21 (1.89–58.12)
2	Two studies; *n* = 240	Rh incompatibility^b^	0.803 (0.342–0.973)	0.680 (0.253–0.921)	2.32 (0.70–9.40)	0.305 (0.04–1.43)	8.96 (0.47–108.94)
3	Two studies; *n* = 446	ABO/Rh incompatibility^c^	0.501 (0.139–0.855)	0.890 (0.670–0.949)	4.26 (1.07–11.93)	0.574 (0.16–0.98)	7.64 (1.09–56.05)

CrI, credible interval; LR+, positive likelihood ratio; LR−, negative likelihood ratio; DOR, diagnostic odds ratio; n, sample size; Rh, Rhesus.

^a^ ABO incompatibility: Mother blood group—O and Rh positive; and neonate blood group—A or B and Rh positive or negative.

^b^ Rh incompatibility: Mother blood group—A, B, AB, or O and Rh negative; and neonate blood group—A, B, AB, or O and Rh positive.

^c^ ABO/Rh incompatibility: Mother blood group—O and Rh positive; and neonate blood group—A or B and Rh positive or negative **OR** Mother blood group—A, B, AB, or O and Rh negative; and neonate blood group—A, B, AB, or O and Rh positive.

For the target condition, the need for DVET, a total of seven studies (*n* = 3,338) (19, 22, 27, 29, 31, 32, 44) were included. In ABO incompatibility settings (three studies; *n* = 2,652) (19, 32, 44), the pooled sensitivity and specificity were 83.6% (95% CrI: 35.8%–99.6%) and 74.5% (95% CrI: 40.3%–92.7%). In Rh incompatibility (two studies; 240 infants) (22, 29), the pooled sensitivity and specificity were 80.3% (95% CrI: 34.2%–97.3%) and 68.0% (95% CrI: 25.3%–92.1%). Whereas for ABO/Rhesus incompatibility (two studies; *n* = 446) (27, 31), the pooled sensitivity and specificity were 50.1% (95% CrI: 13.9%–85.5%) and 89.0% (95% CrI: 67.0%–94.9%) (Table 3 and Supplementary Figure 7).

For the target condition, the need for IVIG (two studies; *n* = 1,005) (19, 45), we could not perform a meta-analysis as only a limited number of studies on each blood group scenario had reported on this outcome.

#### 3.4.1 Subgroup analysis

##### 3.4.1.1 Treatment thresholds

Subgroup analysis was conducted for the need for phototherapy for the studies which used the American Academy of Pediatrics (AAP) threshold chart in the ABO (nine studies, *n* = 3,231) (19, 26, 28, 30, 35, 36, 38, 39) and ABO/Rh incompatibility (six studies, *n* = 7,807) (20, 21, 23, 25, 27) scenarios. Whereas subgroup analyses were performed for studies using National Institute for Health and Care Excellence (NICE) threshold charts in the Rh (two studies, *n* = 483) (10, 29) and ABO/Rh (two studies; *n* = 1,611) (10, 24) incompatibility scenarios. There were no significant differences in the subgroup analyses except for an improvement in sensitivity when using AAP charts for the need for phototherapy in ABO incompatibility scenarios. The sensitivity and specificity were 80.3% (95% CrI: 34.2%–97.3%) and 68.0% (95% CrI: 25.3%–92.1%), respectively (Supplementary Table 3).

##### 3.4.1.2 DAT method

The subgroup analysis based on the method used for DAT measurement (gel vs. tube method) showed no significant difference for the outcomes of phototherapy and DVET in any of the blood group scenarios (Supplementary Table 3).

#### 3.4.2 *Post-hoc* sensitivity analysis

We also performed a *post-hoc* sensitivity analysis by excluding studies with applicability concerns in patient selection (21, 22, 27, 37). For the need for phototherapy, we found no differences from the primary analysis for ABO incompatibility (17 studies, *n* = 10,012) (10, 19, 26, 28, 30, 32–36, 38–43), Rh incompatibility (two studies, *n* = 483) (10, 29), and ABO/Rh incompatibility (seven studies, *n* = 9,076) (10, 20, 23–25, 31) (Supplementary Table 4).

#### 3.4.3 Certainty of evidence

The certainty of evidence for the outcome measure, the need for phototherapy, was very low for the pooled sensitivity in ABO, Rh, and ABO/Rh incompatibility. This was primarily due to inconsistency, followed by the risk of bias and imprecision. In contrast, the certainty of evidence for specificity varied between low to moderate in ABO, Rh, and ABO/Rh incompatibility, mainly due to the risk of bias and inconsistency. For the outcome measure, the need for DVET, the certainty of evidence for sensitivity was very low. For specificity, it remained very low to low across ABO, Rh, and ABO/Rh incompatibility scenarios. Downrating the certainty of evidence was done mainly due to the risk of bias and inconsistency (Table 4).

**Table 4:** GRADE assessment for the certainty of evidence for the effect estimates of the sensitivity and specificity of DAT in predicting the need for phototherapy and DVET.

Target condition	Blood group scenario	DTA measure	No of studies (*n*)		Factors that may decrease certainty of evidence					Rating up the evidence for the ose–response gradient, plausible confounding, and large effect	Test accuracy CoE
				Pooled point estimate	Risk of bias	Indirectness	Inconsistency	Imprecision	Publication bias		
Phototherapy	ABO incompatibility^a^	Sensitivity	18 studies 10,110 neonates	0.56 (0.45–0.68)	Serious^b^	Not serious	Very serious^c^	Serious^d^	None	None	⊕○○○ Very low
		Specificity	18 studies 10,110 neonates	0.84 (0.72–0.91)	Serious^b^	Not serious	Serious^e^	Not serious	None	None	⊕⊕○○ Low
	Rh incompatibility^f^	Sensitivity	Three studies 491 neonates	0.40 (0.12–0.82)	Serious^g^	Not serious	Very serious^h^	Very serious^i^	None	None	⊕○○○ Very low
		Specificity	Three studies 491 neonates	0.90 (0.73–0.95)	Serious^g^	Not serious	Not serious^j^	Not serious	None	None	⊕⊕⊕○ Moderate
	ABO/Rh incompatibility^k^	Sensitivity	Nine studies 9,561 neonates	0.35 (0.19–0.57)	Serious^l^	Not serious	Serious^m^	Serious^n^	None	None	⊕○○○ Very low
		Specificity	Nine studies 9,561 neonates	0.82 (0.62–0.93)	Serious^l^	Not serious	Not serious^o^	Not serious	None	None	⊕⊕⊕○ Moderate
DVET	ABO incompatibility^a^	Sensitivity	Three studies 2,652 neonates	0.84 (0.36–1.00)	Serious^p^	Not serious	Serious^q^	Serious^r^	None	None	⊕○○○ Very low
		Specificity	Three studies 2,652 neonates	0.74 (0.40–0.93)	Serious^p^	Not serious	Not serious^s^	Serious^t^	None	None	⊕⊕○○ Low
	Rh incompatibility^f^	Sensitivity	Two studies 240 neonates	0.80 (0.34–0.97)	Very serious^u^	Not serious	Not serious^v^	Serious^w^	None	None	⊕○○○ Very low
		Specificity	Two studies 240 neonates	0.68 (0.25–0.92)	Very serious^u^	Not serious	Serious^x^	Serious^y^	None	None	⊕○○○ Very low
	ABO/Rh incompatibility^k^	Sensitivity	Two studies 446 neonates	0.50 (0.14–0.85)	Very serious^z^	Not serious	Not serious^aa^	Serious^ab^	None	None	⊕○○○ Very low
		Specificity	Two studies 446 neonates	0.89 (0.67–0.95)	Very serious^z^	Not serious	Not serious^ac^	Not serious	None	None	⊕⊕○○ Low

CrI, credible interval; *n*, sample size; DVET, Double volume exchange transfusion; Rh, Rhesus.

^a^ ABO incompatibility: Mother blood group—O and Rh positive; and neonate blood group—A or B and Rh positive or negative.

^b^ As assessed by QUADAS-2, in the patient selection domain, 6 out of 18 studies had a high risk of bias, and in the reference standard domain, 17 out of 18 studies had a high risk of bias. We did not downgrade further, as blinding to the DAT results might not have had an impact if the guideline threshold had been used.

^c^ For individual studies, the sensitivity varied markedly from 14% to 92%. We downgraded by two levels as we could not perform meta-regression or subgroup analysis for all covariates.

^d^ We downgraded by one level due to the wide CrIs related to the true positives and false negatives.

^e^ For individual studies, the specificity varied from 46% to 98%. We downgraded by one level as we could not perform meta-regression or subgroup analysis for all covariates.

^f^ Rh incompatibility: Mother blood group—A, B, AB, or O and Rh negative; and neonate blood group—A, B, AB, or O and Rh positive.

^g^ As assessed by QUADAS-2, in the patient selection domain, two out of three studies had a high risk of bias, and in the reference standard domain, two out of three studies had a high risk of bias. We did not downgrade further, as blinding to the DAT results might not have had an impact if the guideline threshold had been used.

^h^ For individual studies, the sensitivity varied markedly from 8% to 100%. We downgraded by two levels as we could not perform a meta-regression or subgroup analysis for all covariates.

^i^ We downgraded by two levels due to the wide CrIs related to the true positives and false negatives.

^j^ We did not downgrade as the specificity varied between 88% and 93%.

^k^ ABO/Rh incompatibility: Mother blood group—O and Rh positive; and neonate blood group—A or B and Rh positive or negative OR Mother blood group—A, B, AB, or O and Rh negative; and neonate blood group—A, B, AB, or O and Rh positive.

^l^ As assessed by QUADAS-2, in the patient selection domain, three out of nine studies had a high risk of bias, and in the reference standard domain, seven out of nine studies had a high risk of bias. We did not downgrade further, as blinding to the DAT results might not have had an impact if the guideline threshold had been used.

^m^ For individual studies, sensitivity varied markedly from 12% to 94%. We downgraded by one level as we could not perform a meta-regression or subgroup analysis for all covariates.

^n^ We downgraded by one level due to the wide CrIs related to the true positives and false negatives.

^o^ For individual studies, the specificity varied markedly from 6% to 99%. We did not downgrade as the majority of studies (9 out of 10) had a range from 75% to 99%.

^p^ As assessed by QUADAS-2, in the patient selection domain, one out of three studies had a high risk of bias, and in the reference standard domain, two out of three studies had a high risk of bias. We did not downgrade further, as blinding to the DAT results might not have had an impact if the guideline threshold had been used.

^q^ For individual studies, the sensitivity varied from 66% to 100%. We downgraded by one level as we could not perform a meta-regression or subgroup analysis for all covariates.

^r^ We downgraded by one level due to the wide CrIs related to the true positives and false negatives.

^s^ For individual studies, the specificity varied markedly from 64% to 85%. We did not downgrade.

^t^ We downgraded by one level due to the wide CrIs related to the true negatives and false positives.

^u^ As assessed by QUADAS-2, in the patient selection domain, two out of two studies had a high risk of bias, and in the reference standard domain, one out of two studies had a high risk of bias. We downgraded by two levels.

^v^ Both studies had a sensitivity of 100%, so we did not downgrade.

^w^ We downgraded by one level due to the wide CrIs related to the true positives and false negatives.

^x^ Specificity ranged from 58% to 92%. We downgraded by one level.

^y^ We downgraded by one level due to the wide CrIs related to the true negatives and false positives.

^z^ As assessed by QUADAS-2, in the patient selection domain, two out of two studies had a high risk of bias, and in the reference standard domain, one out of one study had a high risk of bias. We downgraded by two levels.

^aa^ The sensitivity was 50% in both studies.

^ab^ We downgraded by one level due to the wide CrIs related to the true positives and false negatives.

^ac^ The specificity was 88% in one study and 92% in the other. We did not downgrade.

#### 3.4.4 Publication bias

For the outcome measures, the need for phototherapy and DVET, we did not detect publication bias as Deek's plot for asymmetry was not statistically significant (Supplementary Figures 8, 9).

## 4 Discussion

In our systematic review and DTA meta-analysis, we found that the DAT had low sensitivity but moderate specificity in predicting the need for phototherapy in ABO and Rh incompatibility scenarios. The evidence certainty for sensitivity ranged from very low to low, while for specificity, it was low to moderate. For the outcome measure, the need for DVET, the DAT showed varied sensitivity and specificity across ABO and Rh incompatibility scenarios, with the evidence certainty ranging from very low to low for both sensitivity and specificity.

The sensitivity of DAT for predicting the need for phototherapy was 56.1% in ABO incompatibility, 40.4% in Rh incompatibility, and 35.8% in studies that had evaluated either ABO or Rh incompatibility pairs, with only modest changes in specificity in the aforementioned groups, which was moderate. In addition, the predictive interval for Rh and ABO/Rh incompatibility was as low as 14%, indicating that the DAT is a poor predictor of the need for phototherapy in these scenarios. The poor predictive ability of DAT in Rh and ABO/Rh incompatibility could likely be attributed to a false positive rate of approximately 15% due to the routine use of maternal anti-D immunoglobulin in Rh-negative mothers and the passive transfer of these immunoglobulins to infants (46). The specificity of DAT for predicting the need for phototherapy was 83.6%, 89.9%, and 82.5% in ABO, Rh, and ABO/Rh incompatibility, respectively. However, not accounting for other causes of NNH, such as G6PD deficiency and extravasation, likely could have impacted the specificity of DAT. In contrast, the sensitivity and specificity of the DAT for predicting the need for DVET varied markedly across different blood group incompatibility scenarios. For ABO and Rh incompatibility, the sensitivity was above 80%, while the specificity was relatively lower.

To illustrate the practical implications of our study findings, we could consider 1,000 neonates with ABO incompatibility with a 20% probability [based on Gabbay et al. 2023 (20)] of them needing phototherapy for NNH. In this scenario, there would be 243 positive test results. However, 131 (41%) neonates would be falsely diagnosed as having ongoing hemolysis (false positive), whereas, among the 757 negative DAT results, 88 (12%) would be incorrect diagnoses (false negative). This high rate of false positives could lead to delayed discharge, additional investigations, an increased burden on the healthcare system, and heightened parental anxiety. On the other side, false negatives pose a significant risk, as high-risk neonates might be discharged earlier and followed up less frequently, placing the infants at risk of significant hyperbilirubinemia. In addition, the cost implications are considerable. Nevertheless, the moderate specificity of DAT for ABO incompatibility would allow 669 (88%) out of the 757 cases with negative results to have the correct diagnosis (true negatives). This improved specificity may facilitate early discharge and reduce costs.

The studies we included had a moderate risk of bias, especially in the reference standard domain. This was due to the non-blinding of the DAT test results to clinicians, which could significantly influence the need for an intervention. In addition, there was uncertainty in the flow and timing domain due to the poor reporting of the included studies.

Our review has crucial clinical implications, as it is the first comprehensive review addressing the utility of DAT in the management of neonates with blood group incompatibility. Previously, conflicting evidence on the utility of DAT led to varied recommendations by academic bodies. The AAP recommends a routine umbilical cord blood DAT for all mothers with a history of antenatal antibody screen being positive or unknown or with an Rh-negative blood group (1). The NICE advises against using routine umbilical cord blood DATs to predict significant NNH (4). As a DAT has a low sensitivity in most situations, a DAT may not be used as a screening tool for predicting the need for NNH treatment. Our findings imply that a negative DAT result in an incompatibility scenario can reasonably identify neonates who are less likely to require treatment for NNH and may be restricted to at-risk neonates to assess their risk stratification. Moreover, the sensitivity and specificity of a DAT in the prediction of DVET varied markedly with a wide predictive interval and very low to low evidence certainty. Therefore, we do not suggest the use of the DAT as a screening test for predicting severe hyperbilirubinemia requiring DVET.

Despite including several studies, the certainty of evidence remained very low to low for sensitivity and low to moderate for specificity for the need for phototherapy outcome measure, implying the need for future studies with improved study designs.

### 4.1 Strengths and limitations

To the best of our knowledge, this is the first systematic review to assess the predictive ability of DAT for determining the need for an intervention in NNH. We conducted a thorough literature search and performed clinically relevant analyses based on various blood group combinations, including various subgroup and *post-hoc* sensitivity analyses. We adhered to the standards recommended by the Cochrane Screening and Diagnostic Tests Methods group and followed the PRISMA reporting guidelines. We assessed the certainty of evidence as guided by the GRADE working group. However, our study had some limitations. Initially, we planned to include all blood group combinations in the meta-analyses, but we restricted the analysis to clinically relevant incompatibility scenarios. We were unable to do many of the pre-planned subgroup analyses such as based on gestational age and DAT strength due to the non-availability of data. We could not evaluate the effect of the updated AAP guidelines on phototherapy and DVET on the sensitivity and specificity of DAT, as none of the included studies evaluated these thresholds. Finally, we did not account for ethnic differences or consider other causes of significant hyperbilirubinemia.

## 5 Conclusions

In ABO and Rh incompatibility, DAT probably has moderate specificity but low sensitivity for predicting the need for phototherapy. For the need for DVET, DAT is possibly a poor predictor as the sensitivity and specificity varied markedly across blood groups with wide predictive intervals. Thus, we do not suggest the use of DAT as a screening test for predicting hyperbilirubinemia requiring either phototherapy or DVET.

## Data Availability

The raw data supporting the conclusions of this article will be made available by the authors, without undue reservation.
