# Supplementary material for: Direct antiglobulin test for the prediction of neonatal hyperbilirubinemia needing an intervention: a systematic review and diagnostic test accuracy meta-analysis
Source: Front Pediatr. 2025 Jan 28;12:1475623. doi: 10.3389/fped.2024.1475623 (PMC11811085; doi:10.3389/fped.2024.1475623)
Supplement: Supplementary file 1 [file Datasheet1.docx]

**Supplementary Material:**

**Tables**

**Supplementary Table 1**: Literature search strategy

**Supplementary Table 2**: List of excluded studies and valid reasons for exclusion

**Supplementary Table 3**: Subgroup analysis for treatment threshold charts and DAT measurement methods as per blood group combinations.

**Supplementary Table 4**: Sensitivity analysis after exclusion of studies with applicability concerns in patient selection domain

**Figures**

**Supplementary Figure 1**: Forest plot depicting sensitivity and specificity of studies utilizing DAT as an index test to predict the need for phototherapy in ABO, ABO / Rh, Rh incompatibility, and all blood group combination settings.

**Supplementary Figure 2**: Summary receiver operating characteristic (SROC) plot of studies utilizing DAT as an index test to predict the need for phototherapy in ABO, ABO / Rh, Rh incompatibility, and all blood group combination settings.

**Supplementary Figure 3**: Forest plot depicting sensitivity and specificity of studies utilizing DAT as an index test to predict the need for DVET in ABO, ABO / Rh, Rh incompatibility, and all blood group combination settings.

**Supplementary Figure 4**: Summary receiver operating characteristic (SROC) plot of studies utilizing DAT as an index test to predict the need for DVET in ABO, ABO / Rh, Rh incompatibility, and all blood group combination settings.

**Supplementary Figure 5**: Forest plot depicting sensitivity and specificity of studies utilizing DAT as an index test to predict the need for IVIG in ABO incompatibility, and all blood group combination settings.

**Supplementary Figure 6**: Summary receiver operating characteristic (SROC) plot of studies utilizing DAT as an index test to predict the need for IVIG in ABO incompatibility, and all blood group combination settings.

**Supplementary Figure 7**: Summary Receiver Operating Characteristic (SROC) plot of studies as per blood group combination for DAT as index test to predict the need for DVET.

**Supplementary Figure 8**: Funnel plot of the log Diagnostic Odds Ratio (DOR) for studies with need for phototherapy as outcome measure.

**Supplementary Figure 9**: Funnel plot of the log Diagnostic Odds Ratio (DOR) for studies with need for DVET as outcome measure.

**Appendix 1:** Narrative review of studies not synthesized in a meta-analysis.

**Supplementary Table 1:** Literature search strategy

Database(s): Ovid MEDLINE(R), Ovid MEDLINE(R) Daily and Epub Ahead of Print, In-Process & Other Non-Indexed Citations

 From inception to February 2024

MEDLINE

| **#** | **Searches** |
| --- | --- |
| 1 | Direct antiglobulin test*.mp. or exp Coombs Test/ |
| 2 | (Test* adj3 (globulin* or antiglobulin* or coomb* or dat)).ab,ti. |
| 3 | 1 or 2 |
| 4 | exp Hyperbilirubinemia, Neonatal/ |
| 5 | ((neonat* or infan* or newborn* or bab*) adj20 (hyperbilirubinemia or bilirubin* or phototherap*)).ab,ti. |
| 6 | 4 or 5 |
| 7 | 3 and 6 |
| 8 | Infant, Newborn/ |
| 9 | (neonat* or infan* or newborn* or bab*).ab,ti. |
| 10 | 8 or 9 |
| 11 | 3 and 10 |
| 12 | [phototherapy.mp](http://phototherapy.mp). or exp Phototherapy/ |
| 13 | 11 and 12 |
| 14 | 7 or 13 |

EMBASE: From inception to February 2024

| **#** | **Searches** |
| --- | --- |
| 1 | Direct antiglobulin test*.mp. or exp Coombs Test/ |
| 2 | (Test* adj3 (globulin* or antiglobulin* or coomb* or dat)).ab,ti. |
| 3 | Direct Antiglobulin Titer.mp. |
| 4 | 1 or 2 or 3 |
| 5 | exp Hyperbilirubinemia, Neonatal/ |
| 6 | ((neonat* or infan* or newborn* or bab*) adj20 (hyperbilirubinemia or bilirubin* or phototherap*)).ab,ti. |
| 7 | Infant, Newborn/ |
| 8 | [jaundice.mp](http://jaundice.mp). or exp Jaundice, Neonatal/ or exp Jaundice/ |
| 9 | 7 and 8 |
| 10 | 5 or 6 or 9 |
| 11 | 4 and 10 |
| 12 | exp animals/ not humans.sh. |
| 13 | 11 and 12 |

**Supplementary Table 2:** List of excluded studies with valid reasons for exclusion

|  | Author, Year | Title, Journal | Reason for exclusion |
| --- | --- | --- | --- |
| 1 | Lieberman 2020 (1) | Impact of red blood cell alloimmunization on fetal and neonatal outcomes: A single centre cohort study.  Transfusion | Data for 2x 2 table could not be retrieved, Outcomes not reported |
| 2 | Bhutani 2013 (2) | Predischarge Screening for Severe Neonatal Hyperbilirubinemia Identifies Infants Who Need Phototherapy  The Journal of Pediatrics | Data for 2x 2 table could not be retrieved, review specific outcomes not reported. |
| 3 | Dillon 2010 (3) | Has anti-D prophylaxis increased the rate of positive direct antiglobulin test results and can the direct antiglobulin test predict need for phototherapy in Rh/ABO incompatibility.  Journal of Paediatrics and Child Health | Data for 2x 2 table could not be retrieved, review specific outcomes not reported. |
| 4 | Lehlimi 2019(4) | Fetal-maternal incompatibility in ABO system.  Journal of Pediatrics and Childcare | Data for 2x 2 table could not be retrieved, review specific outcomes not reported. |
| 5 | Cetinkaya 2021(5) | Nucleated Red blood cell levels in the diagnosis of patients with indirect hyperbilirubinemia.  Minerva Pediatrics | Data for 2x 2 table could not be retrieved, review specific outcomes not reported. |
| 6 | Pocock, 2017(6) | Abstract: Maternal red cell antibodies: incidence and outcomes from a single centre in North London.  British Journal of Haematology | Data for 2x 2 table could not be retrieved, review specific outcomes not reported. |
| 7 | Peeters 2016 (7) | Post-test probability for neonatal hyperbilirubinemia based on umbilical cord blood bilirubin, direct antiglobulin test, and ABO compatibility results.  European journal of Pediatrics | Data for 2x 2 table could not be retrieved, review specific outcomes not reported. |
| 8 | Kukreja 2014(8) | Abstract: Significance of the positive direct antibody test in neonatal ABO incompatibility,  Pediatric blood and Cancer | Data for 2x 2 table could not be retrieved, review specific outcomes not reported. |
| 9 | Rao L 2012 (9) | Abstract: The clinical spectrum of ABO incompatibility and hemolytic disease in the newborn.  Blood | Data for 2x 2 table could not be retrieved, review specific outcomes not reported. |
| 10 | Larson 2017(10) | Abstract: Utility of cord blood testing in O positive mothers | Data for 2x 2 table could not be retrieved, review specific outcomes not reported. |
| 11 | Howard 1998 (11) | Consequences for fetus and neonate of maternal red cell allo-immunisation.  *Arch Dis Child Fetal Neonatal Ed* | Data for 2x 2 table could not be retrieved, review specific outcomes not reported. |
| 12 | Vilambil 2017 (12) | Immunohaematological profile of maternal antibody-mediated abo haemolytic disease of foetus and newborn.  J. Evolution Med. Dent. Sci | Data for 2x 2 table could not be retrieved, review specific outcomes not reported. |
| 13 | Elsaie 2020 (13) | Comparison of end-tidal carbon monoxide measurements with direct antiglobulin tests in the management of neonatal hyperbilirubinemia  Journal of Perinatology | Data for 2x 2 table could not be retrieved, review specific outcomes not reported. |
| 14 | Zhang 2020 (14) | A clinical prediction rule for acute bilirubin  encephalopathy in neonates with extreme  hyperbilirubinemia Medicine | Data for 2x 2 table could not be retrieved, review specific outcomes not reported. |
| 15 | Tiras 2020 (15) | Role of Cord Blood Carboxyhemoglobin in Detecting Significant Hyperbilirubinemia in Term Neonates with ABO Alloimmunization.  American journal of perinatology. | Data for 2x 2 table could not be retrieved, review specific outcomes not reported. |
| 16 | Bel Hadj 2019 (16) | Ictère hémolytique par incompatibilité ABO : Le type du groupe sanguin du nouveau-né constitue-t-il un facteur de risque?  La tunisie medicale | Data for 2x 2 table could not be retrieved, review specific outcomes not reported. |
| 17 | Margolis 2019 (17) | Abstract: Direct Antiglobulin Titer (Dat) Strength And Hyperbilirubinemia In Canadian Infants  Pediatrics and Child Health | Data for 2x 2 table could not be retrieved, review specific outcomes not reported. |
| 18 | Schutzman 2016 (18) | Carboxyhemoglobin levels as a predictor of risk for significant hyperbilirubinemia in African-American DAT+ infants  Journal of Perinatology | Data for 2x 2 table could not be retrieved, review specific outcomes not reported. |
| 19 | Oztekin 2013 (19) | Is the strength of direct antiglobulin test important for the duration of phototherapy?  J Matern Fetal Neonatal Med, | Data for 2x 2 table could not be retrieved, review specific outcomes not reported. |
| 20 | Arévalo 1992 (20) | Hemolytic disease of the newborn due to ABO incompatibility. A predictive test. Enfermedad hemolitica del recien nacido por incompatibilidad ABO Prueba predictiva. | Data for 2 x 2 table couldn’t be retrieved |
| 21 | Kirkman 1977 (21) | Further evidence for a racial difference in frequency  of ABO hemolytic disease  The Journal of PEDIATRICS | Data for 2x 2 table could not be retrieved, review specific outcomes not reported. |

| **Supplementary Table 3:** Subgroup analysis for treatment threshold charts and DAT measurement methods as per blood group combinations**.** | | | | | |
| --- | --- | --- | --- | --- | --- |
| Target Condition | Blood Group Setting | Subgroup analysis | Studies/ Participants | Sensitivity  (95% CrI) | Specificity  (95% CrI) |
| Phototherapy | ABO incompatibility ^a^ | Threshold- AAP | 9 studies; n=3231 | 0.803 (0.342, 0.973) | 0.680 (0.253, 0.921) |
|  |  | DAT method- Gel | 4 studies; n=875 | 0.609 (0.405, 0.797) | 0.723 (0.494, 0.875) |
|  |  | DAT method-Tube | 3 studies; n=493 | 0.508 (0.184, 0.843) | 0.755 (0.459, 0.902) |
|  |  |  |  |  |  |
|  | Rh incompatibility ^b^ | Threshold -NICE | 2 studies; n=483 | 0.170 (0.064, 0.416) | 0.907 (0.763, 0.948) |
|  |  | DAT method- Gel | 2 studies; n=259 | 0.480 (0.131, 0.882) | 0.843 (0.490, 0.940) |
|  |  |  |  |  |  |
|  | ABO / Rh incompatibility ^c^ | Threshold -AAP | 6 studies; n=7807 | 0.414 (0.239, 0.614) | 0.781 (0.580, 0.893) |
|  |  | Threshold - NICE | 2 studies; n-1611 | 0.235 (0.108, 0.545) | 0.881 (0.564, 0.971) |
|  |  | DAT method- Gel | 3 studies; n=712 | 0.284 (0.144, 0.538) | 0.834 (0.675, 0.911) |
|  |  |  |  |  |  |
| DVET | ABO incompatibility ^a^ | Insufficient Data | - | - | - |
|  | Rh incompatibility ^b^ | Insufficient Data | - | - | - |
|  | ABO / Rh incompatibility ^c^ | Insufficient Data | - | - | - |

Abbreviations: CrI: credible interval, AAP: American Academy of Pediatrics, NICE: The National Institute for Health and Care Excellence, n: sample size, DAT: direct antiglobulin test, DVET: Double volume exchange transfusion, Rh: Rhesus

^a.^ ABO incompatibility: Mother blood group- O; Rh positive and neonate blood group - A or B; Rh positive or negative

^b.^ Rh incompatibility: Mother blood group- A, B, AB, O ; Rh negative and neonate blood group - A, B, AB, O ; Rh positive.

^c.^ ABO / Rh incompatibility: Mother blood group- O; Rh positive and neonate blood group- A or B ; Rh positive or negative OR Mother blood group- A, B, AB, O ; Rh negative and neonate blood group- A, B, AB, O ; Rh positive

**Supplementary Table 4:** Sensitivity analysis after exclusion of studies with applicability concerns in patient selection domain.

| **Studies/Participants** | | **Blood Group setting** | **Sensitivity**  **(95% CrI)** | **Specificity**  **(95% CrI)** |
| --- | --- | --- | --- | --- |
| Phototherapy | | | | |
| 1 | 17 studies; n = 10012 | ABO incompatibility ^a^ | 0.558 (0.421, 0.687) | 0.847 (0.733, 0.918) |
| 2 | 2 studies; n = 483 | Rh incompatibility ^b^ | 0.188 (0.061, 0.529) | 0.907 (0.684, 0.957) |
| 3 | 7 studies; n = 9076 | ABO / Rh incompatibility ^c^ | 0.273 (0.179, 0.403) | 0.901 (0.790, 0.953) |

Abbreviations: CrI: credible interval, n: sample size, DVET: Double volume exchange transfusion. Rh: Rhesus

^a.^ ABO incompatibility: Mother blood group- O; Rh positive and neonate blood group - A or B; Rh positive or negative

^b.^ Rh incompatibility: Mother blood group- A, B, AB, O ; Rh negative and neonate blood group - A, B, AB, O ; Rh positive.

^c.^ ABO / Rh incompatibility: Mother blood group- O; Rh positive and neonate blood group- A or B ; Rh positive or negative OR Mother blood group- A, B, AB, O ; Rh negative and neonate blood group- A, B, AB, O ; Rh positive

**Supplementary Figure 1**: Forest plot depicting sensitivity and specificity of studies utilizing DAT as an index test to predict the need for phototherapy in ABO, ABO / Rh, Rh incompatibility, and all blood group combination settings.


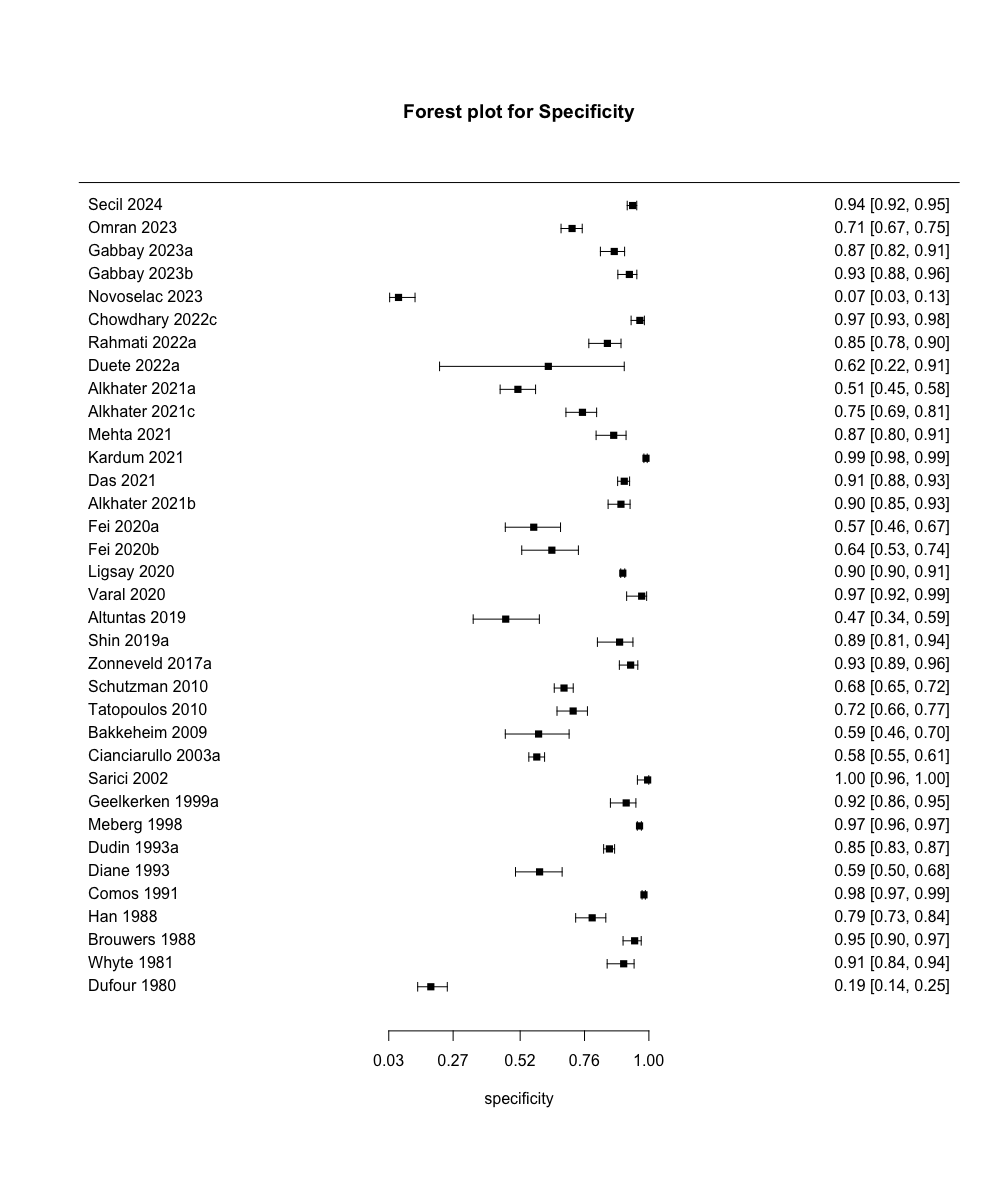


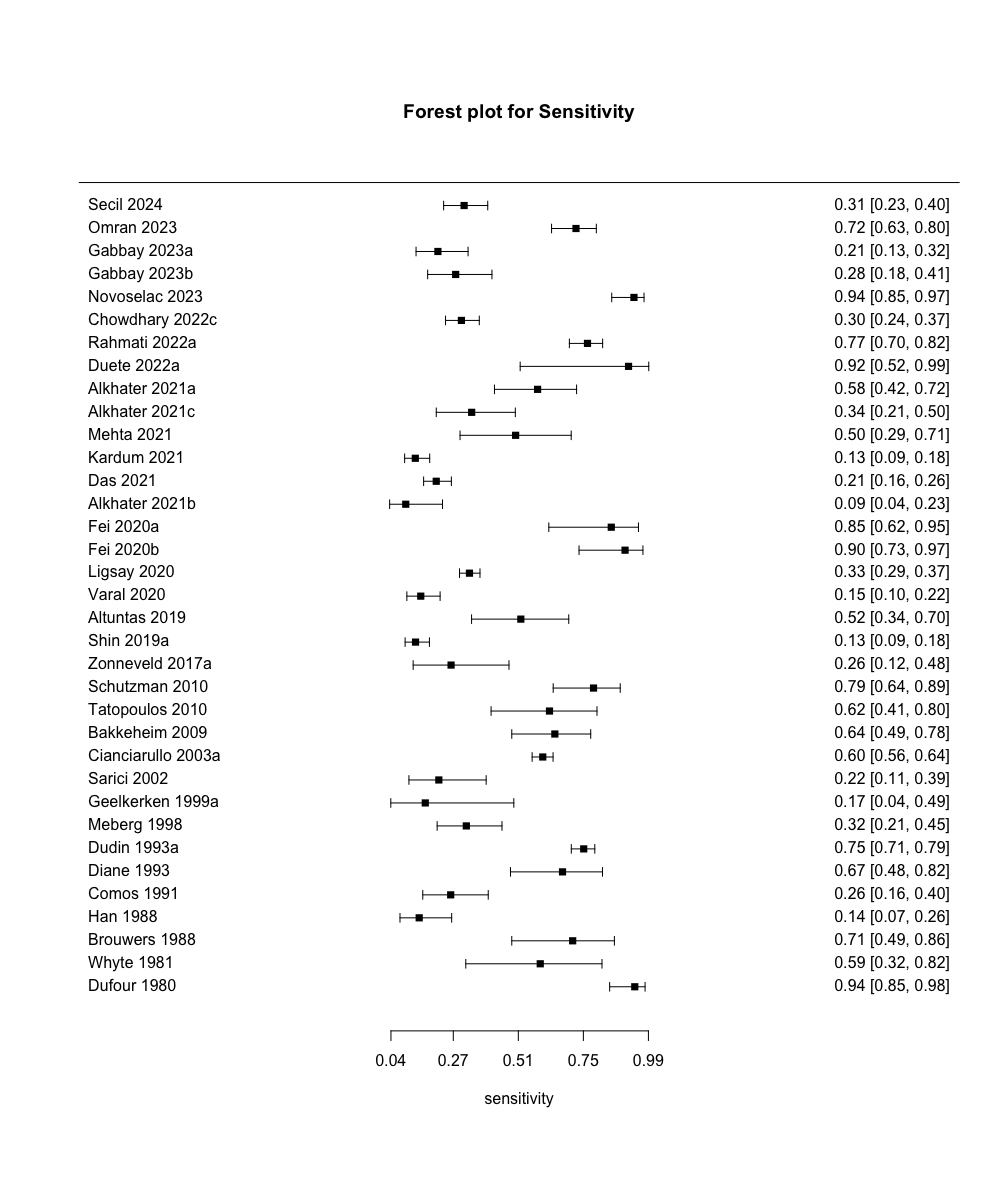


| 1 | Secil 2024 |
| --- | --- |
| 2 | Omran 2023 |
| 3 | Gabbay 2023a |
| 4 | Gabbay 2023b |
| 5 | Novoselac 2023 |
| 6 | Chowdhary 2022c |
| 7 | Rahmati 2022a |
| 8 | Duete 2022a |
| 9 | Alkhater 2021a |
| 10 | Alkhater 2021c |
| 11 | Mehta 2021 |
| 12 | Kardum 2021 |
| 13 | Das 2021 |
| 14 | Alkhater 2021b |
| 15 | Fei 2020a |
| 16 | Fei 2020b |
| 17 | Ligsay 2020 |
| 18 | Varal 2020 |
| 19 | Altuntas 2019 |
| 20 | Shin 2019a |
| 21 | Zonneveld 2017a |
| 22 | Schutzman 2010 |
| 23 | Tatopoulos 2010 |
| 24 | Bakkeheim 2009 |
| 25 | Cianciarullo 2003a |
| 26 | Sarici 2002 |
| 27 | Geelkerken 1999a |
| 28 | Meberg 1998 |
| 29 | Dudin 1993a |
| 30 | Diane 1993 |
| 31 | Comos 1991 |
| 32 | Han 1988 |
| 33 | Brouwers 1988 |
| 34 | Whyte 1981 |
| 35 | Dufour 1980 |

**Supplementary Figure 2:** Summary receiver operating characteristic (SROC) plot of studies utilizing DAT as an index test to predict the need for phototherapy in ABO, ABO/Rh, Rh incompatibility, and all blood group combination settings.


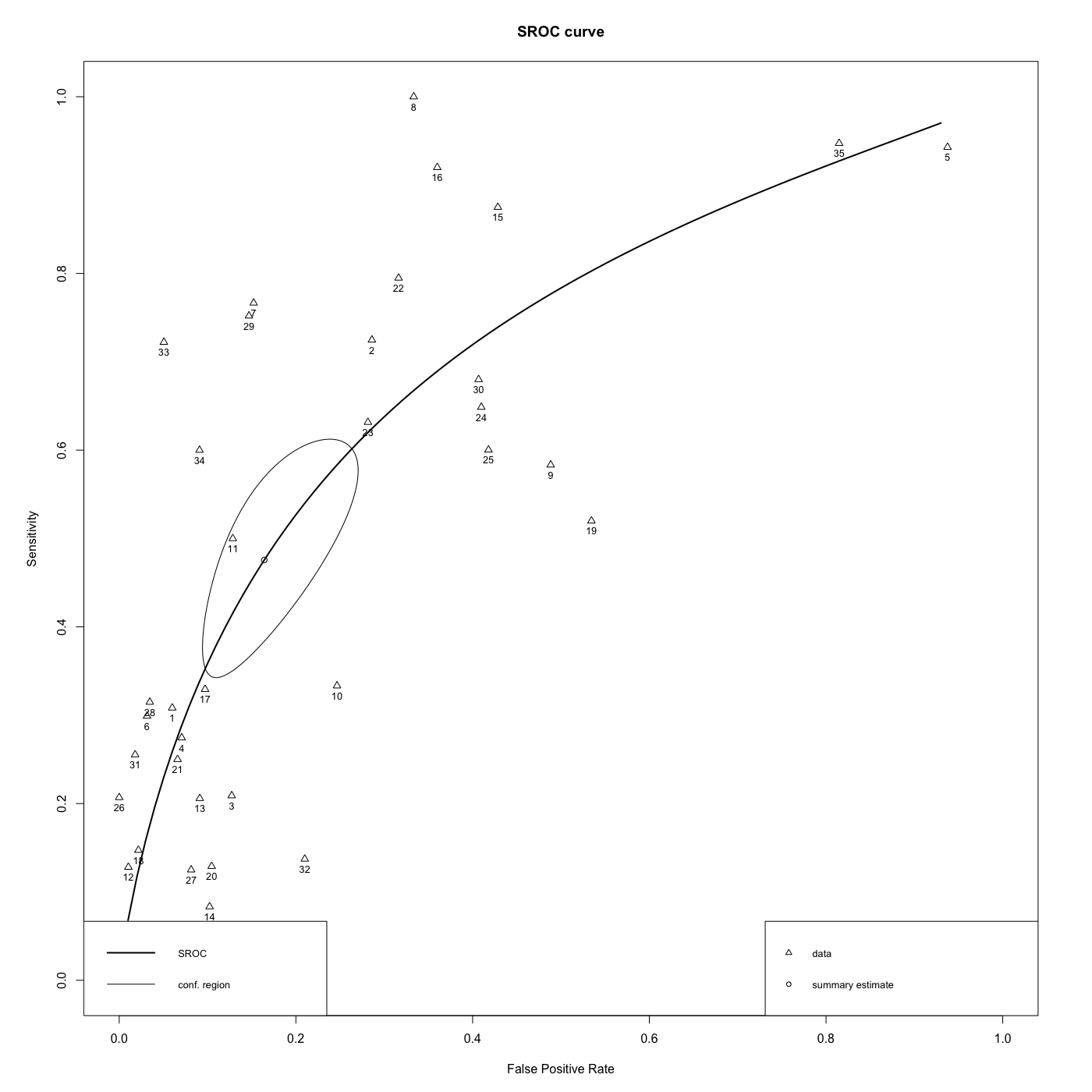


**Supplementary Figure 3:** Forest plot depicting sensitivity and specificity of studies utilizing DAT as an index test to predict the need for DVET in ABO, ABO / Rh, Rh incompatibility, and all blood group combination settings.


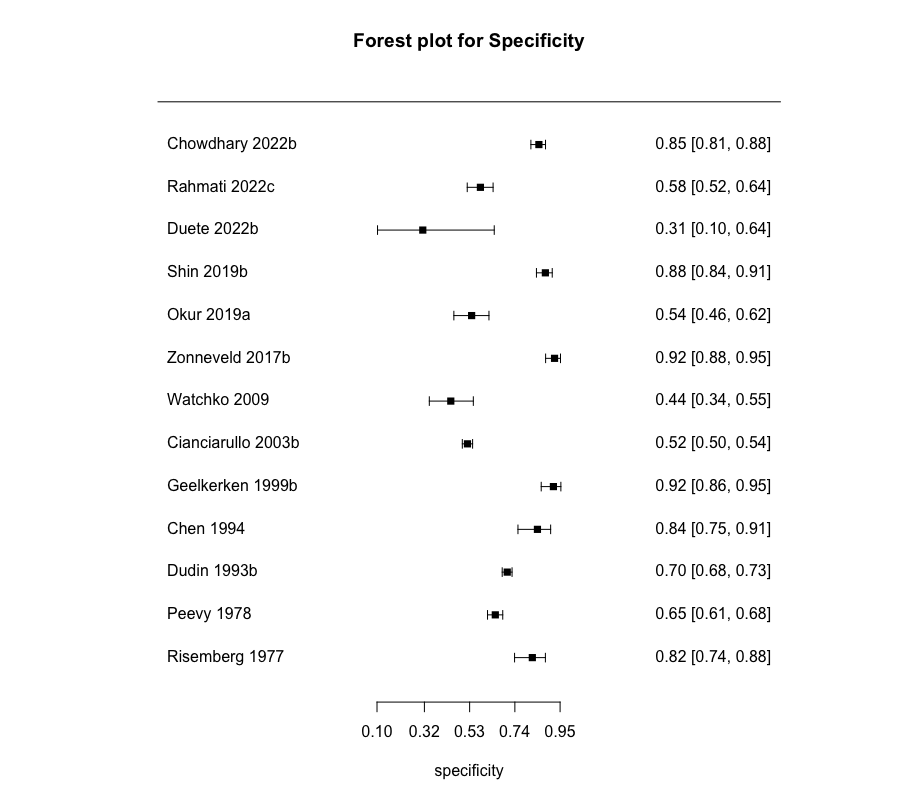


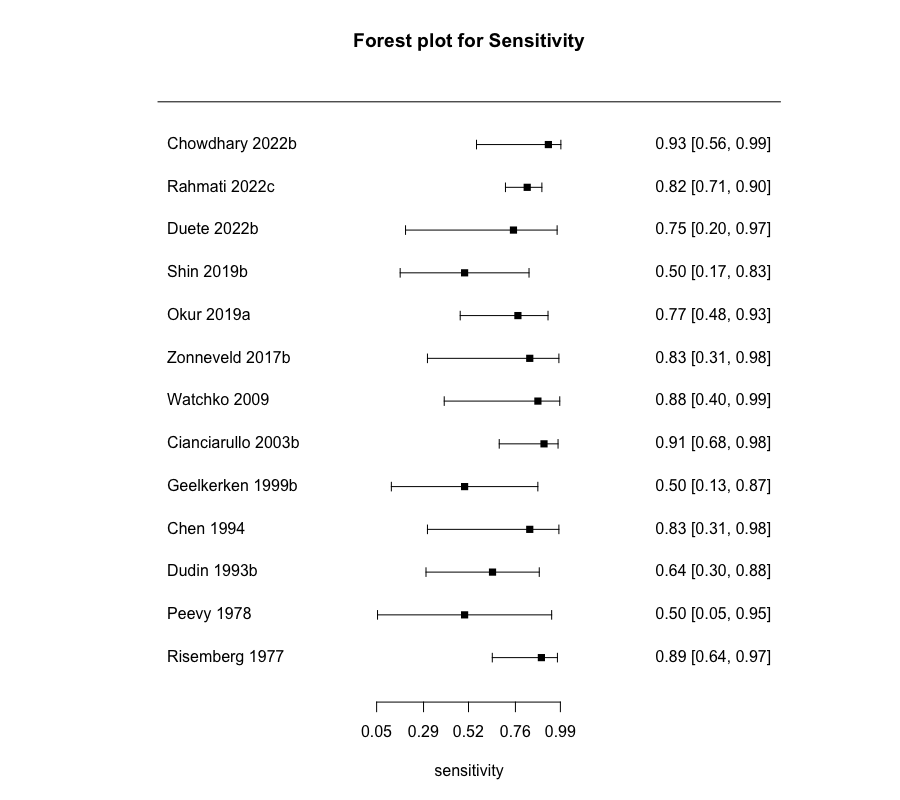


**Supplementary Figure 4:** Summary receiver operating characteristic (SROC) plot of studies utilizing DAT as an index test to predict the need for DVET in ABO, ABO/Rh, Rh incompatibility, and all blood group combination settings.


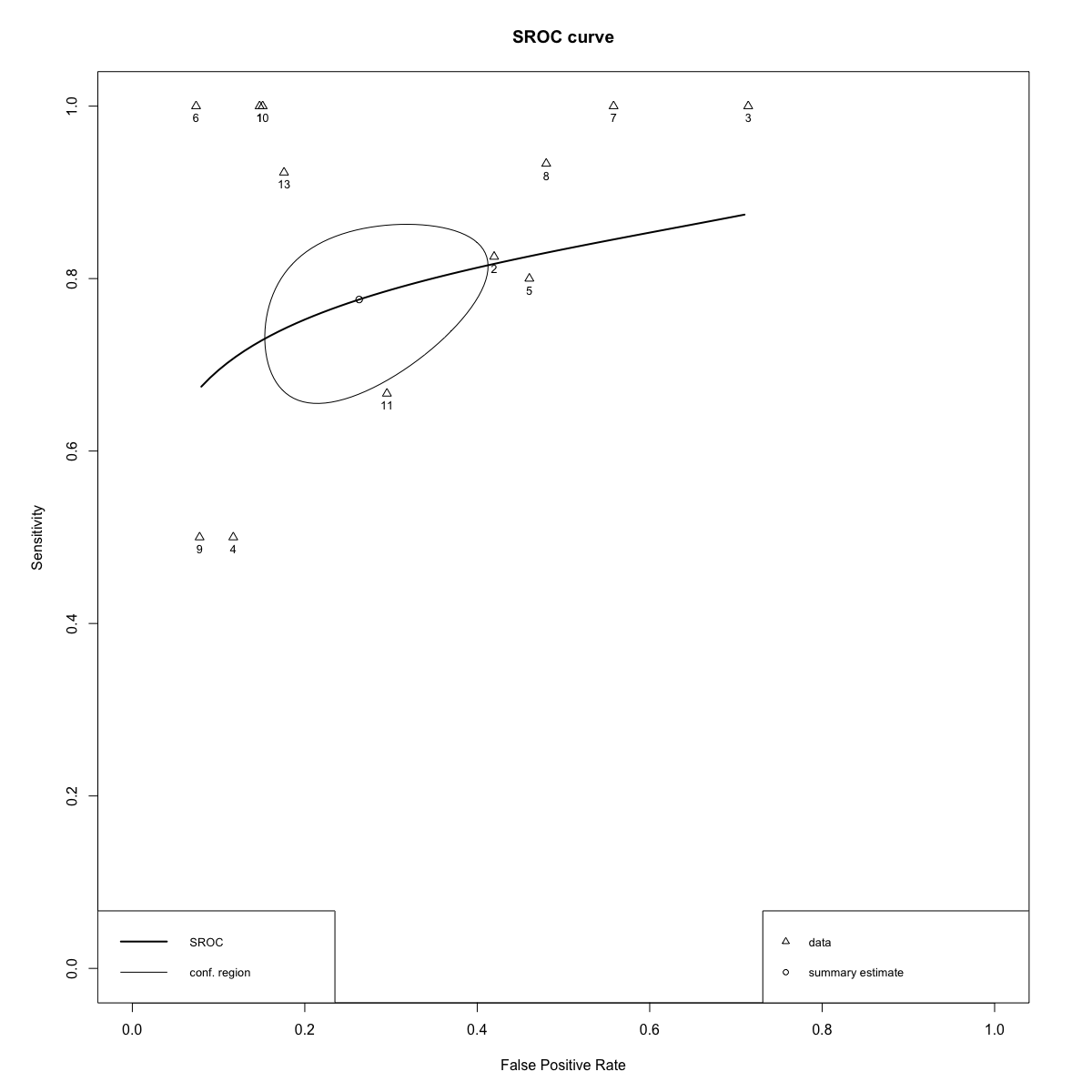


Figure : SROC plot of all studies with need for DVET as target condition

| 1 | Chowdhary 2022b |
| --- | --- |
| 2 | Rahmati 2022c |
| 3 | Duete 2022b |
| 4 | Shin 2019b |
| 5 | Okur 2019a |
| 6 | Zonneveld 2017b |
| 7 | Watchko 2009 |
| 8 | Cianciarullo 2003b |
| 9 | Geelkerken 1999b |
| 10 | Chen 1994 |
| 11 | Dudin 1993b |
| 12 | Peevy 1978 |
| 13 | Risemberg 1977 |

**Supplementary Figure 5**: Forest plot depicting sensitivity and specificity of studies utilizing DAT as an index test to predict the need for IVIG in ABO incompatibility, and all blood group combination settings.


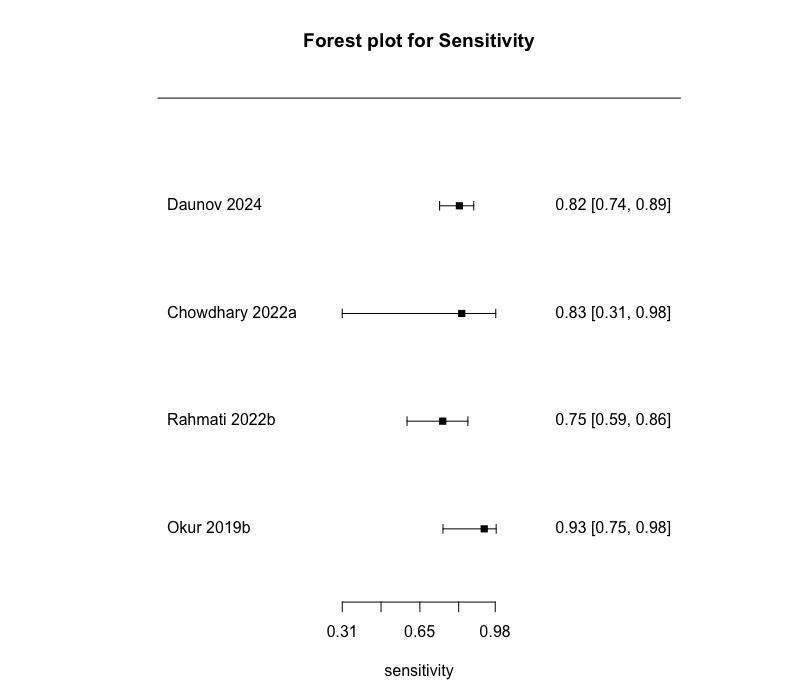


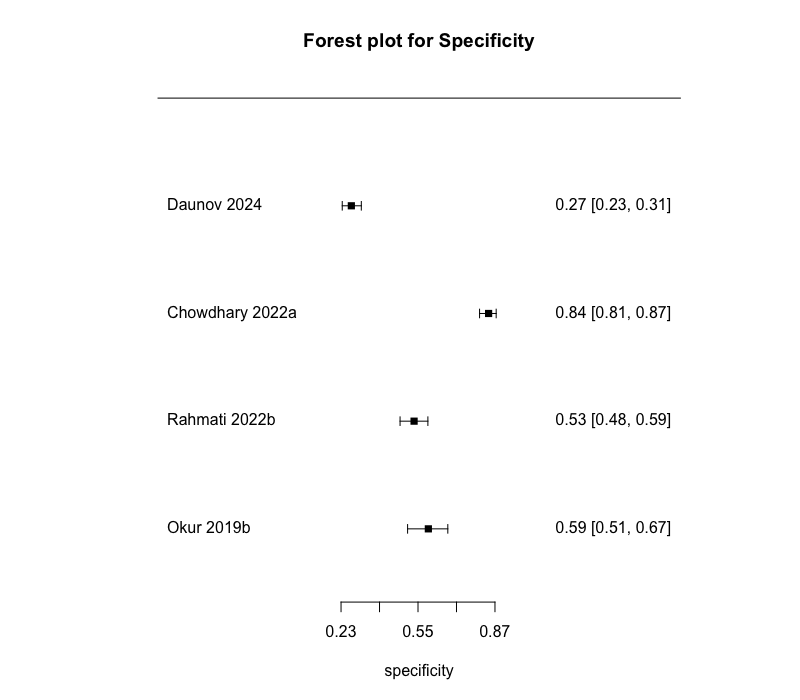


**Supplementary Figure 6:** Summary receiver operating characteristic (SROC) plot of studies utilizing DAT as an index test to predict the need for IVIG in ABO incompatibility, and all blood group combination settings.


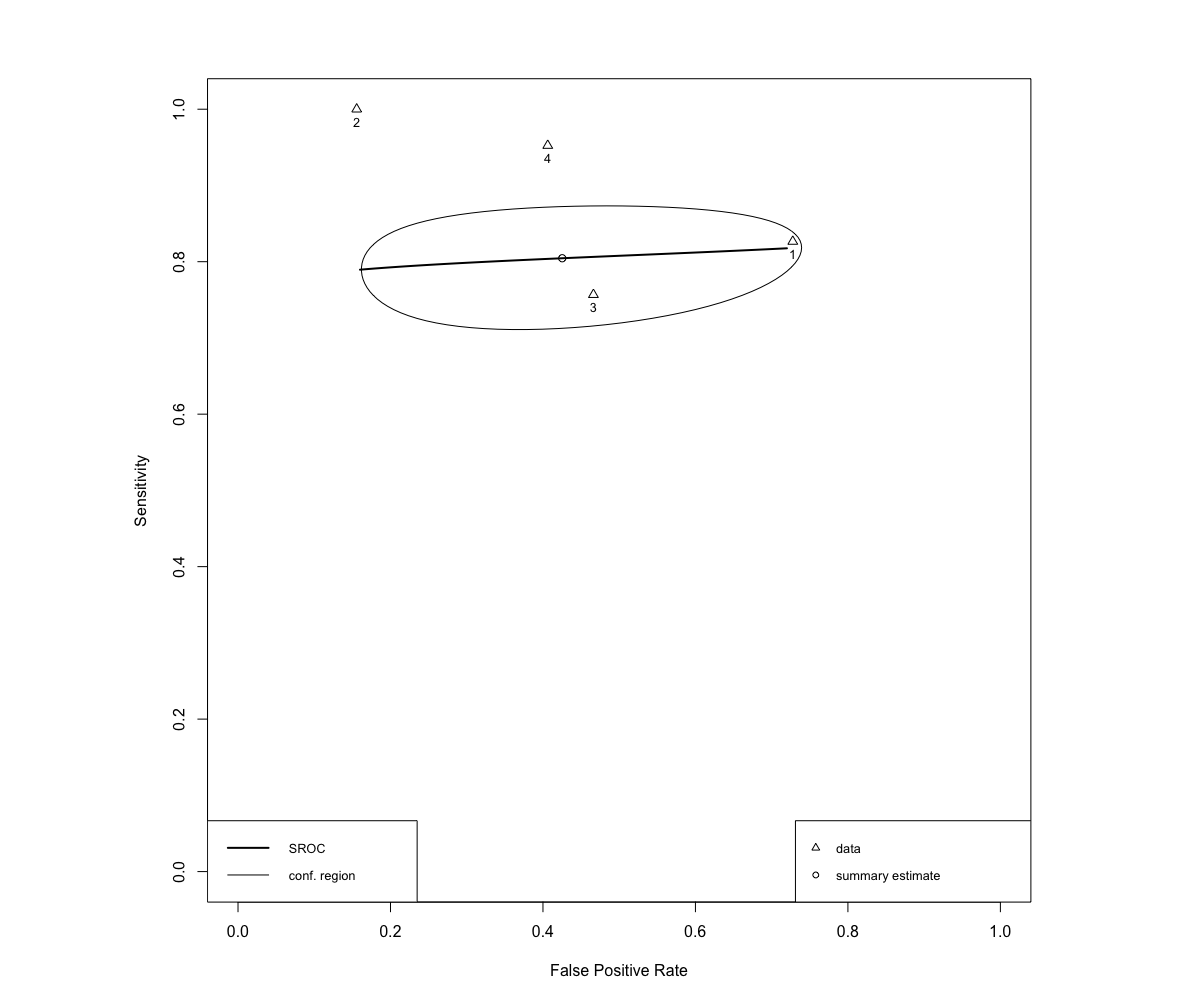


| 1 | Chowdhary 2023 |
| --- | --- |
| 2 | Okur 2019 |
| 3 | Rahmati 2022 |
| 4 | Daunov 2024 |

**Supplementary Figure 7:** Summary Receiver Operating Characteristic (SROC) plot of studies as per blood group combination for DAT as an index test to predict the need for DVET. A. SROC plot for ABO incompatibility, B. SROC plot for Rh incompatibility, C. SROC plot for ABO/Rh incompatibility. Each study is represented by a circle. Small circle with black shade (A) and small triangle (B) and (C) denote the combined sensitivity and specificity. The figure also shows the 95% credible region (red line for A, grey shaded area for B and C) and the 95% prediction region (black line for A, dashed line for B and C).

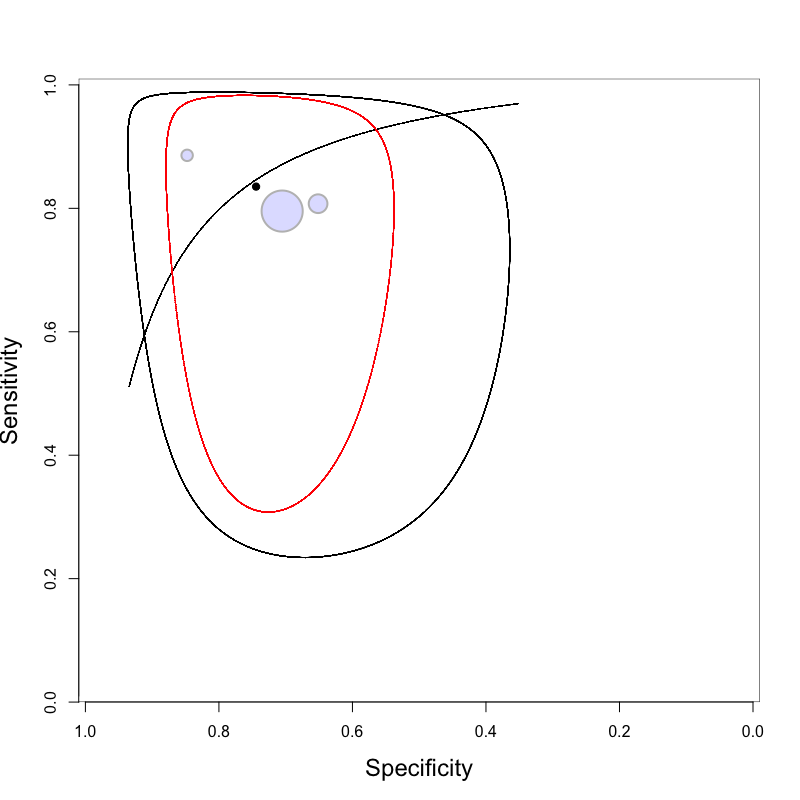

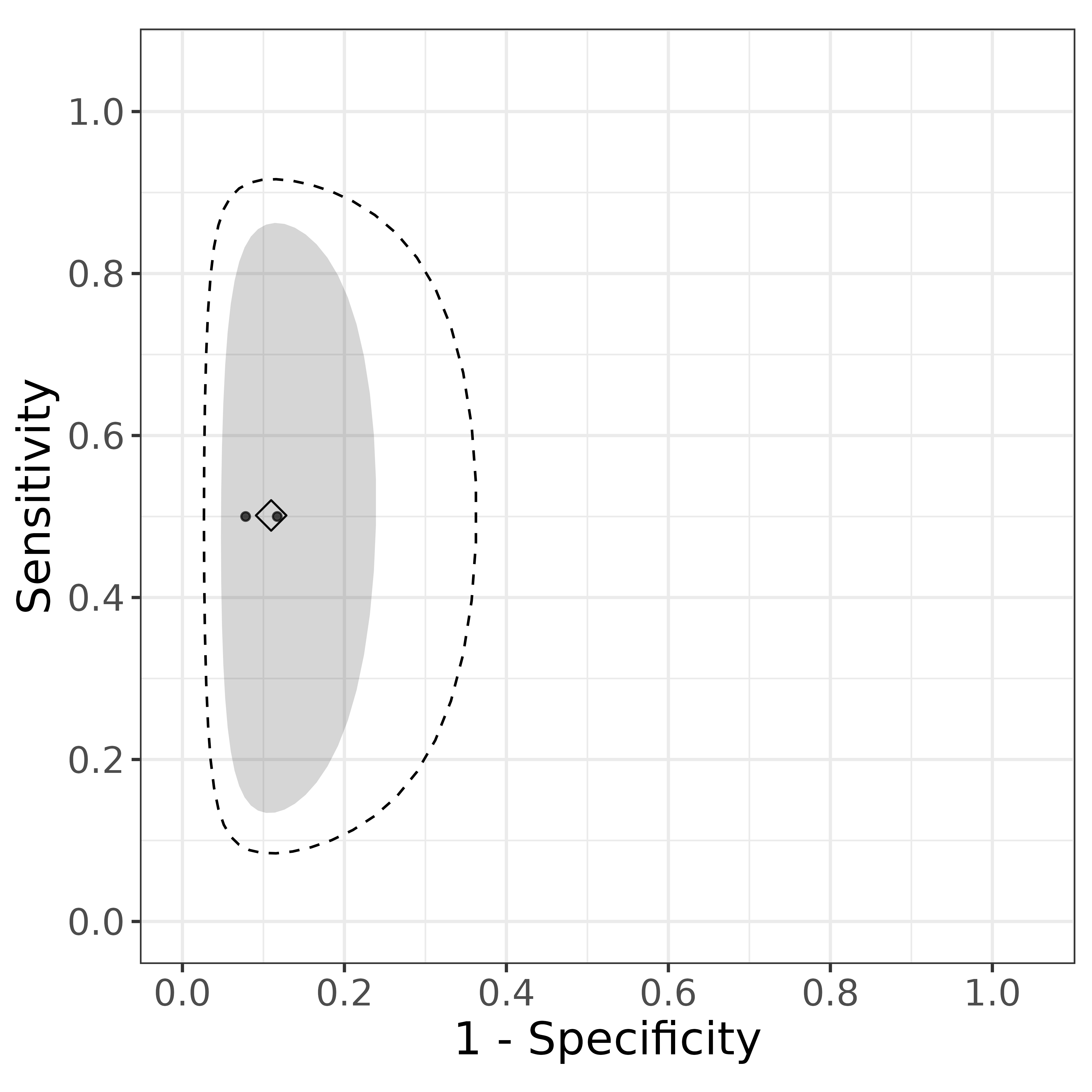


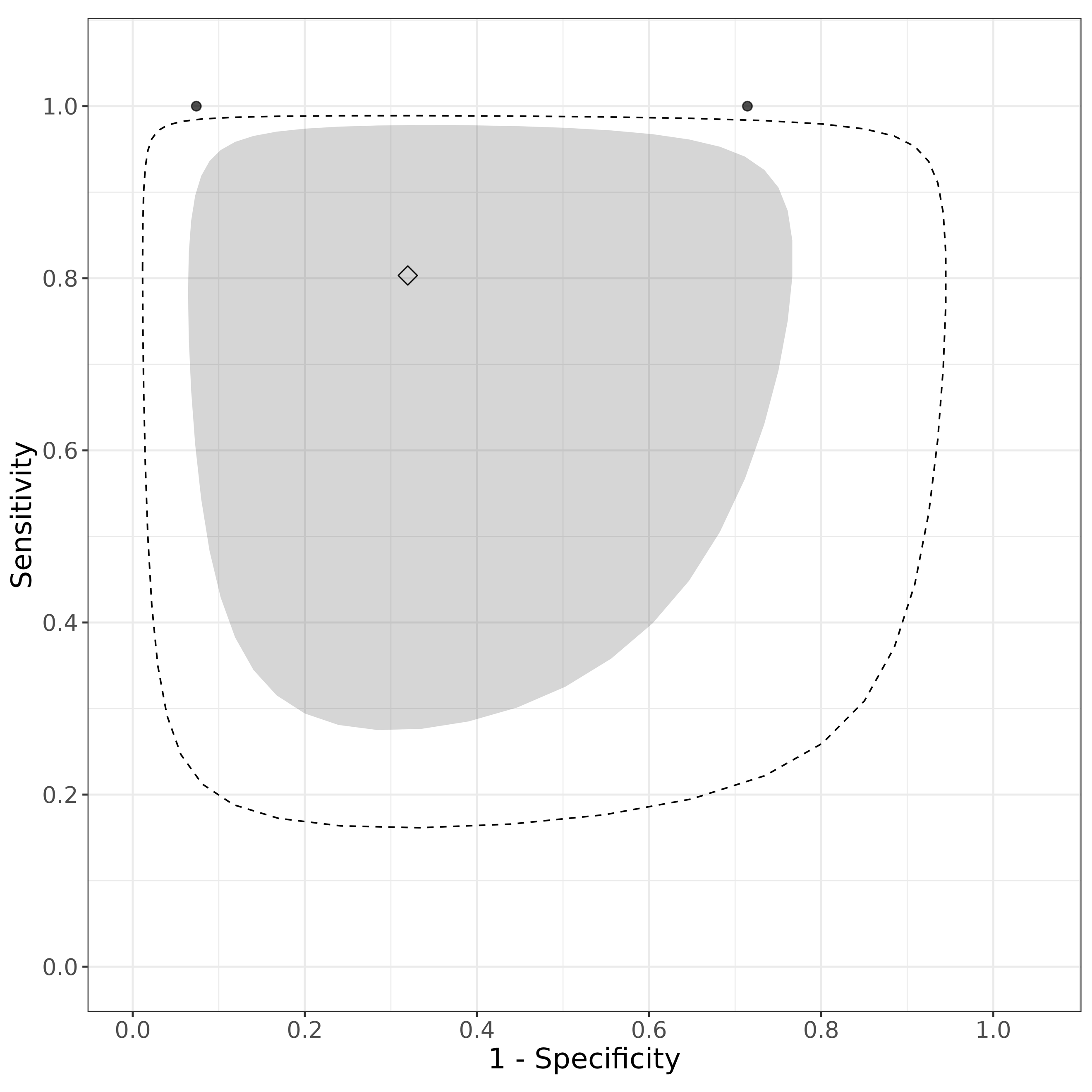


**Supplementary Figure 8**: Funnel plot of the log Diagnostic Odds Ratio (DOR) against the inverse of the square root of the effective sample size, with pseudo 95% confidence intervals for studies with need for phototherapy as outcome measure.


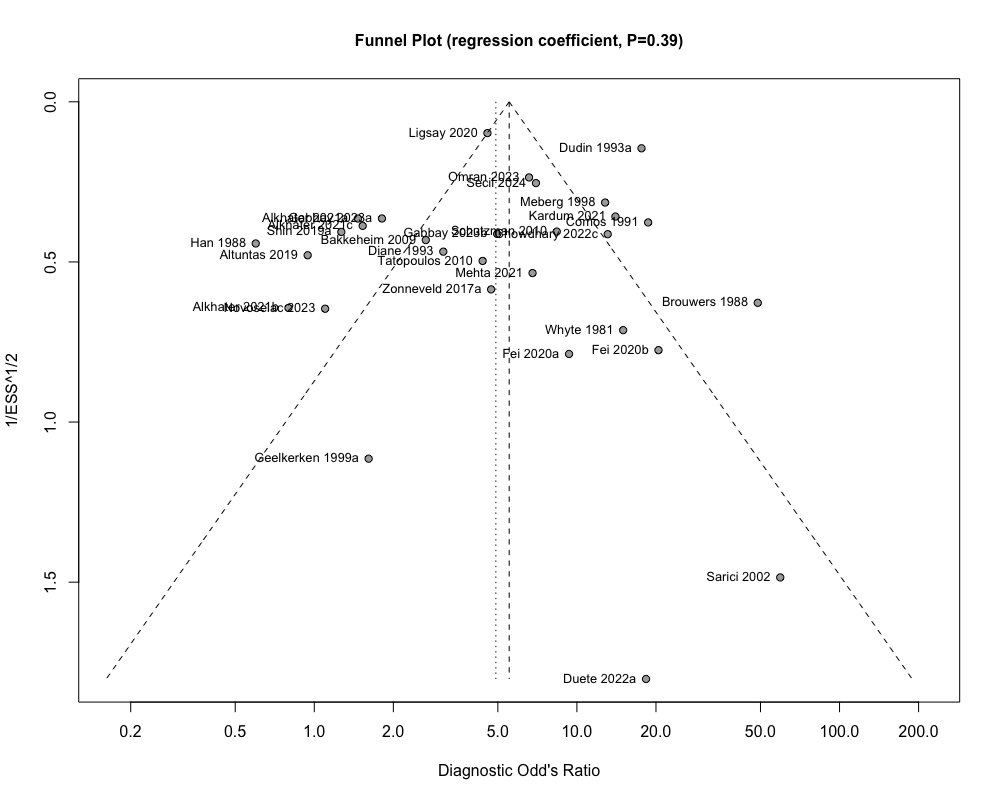


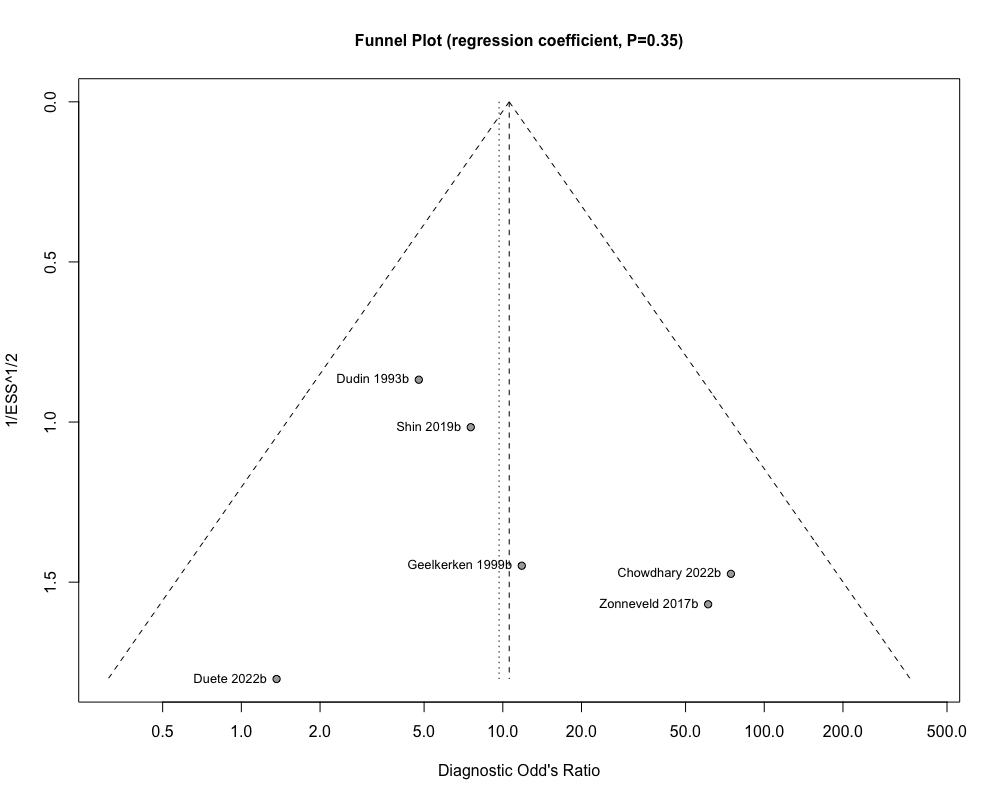


**Supplementary Figure 9**: Funnel plot of the log Diagnostic Odds Ratio (DOR) against the inverse of the square root of the effective sample size, with pseudo 95% confidence intervals for studies with need for DVET as outcome measure.

| **Appendix 1:** Narrative review of studies not synthesized in the meta-analysis. | | | | | | | | |
| --- | --- | --- | --- | --- | --- | --- | --- | --- |
| **No** | **Author**  **Study Design**  **Country** | **Sample Size** | **Gestation**  **(mean/**  **median)** | **Blood Group settings** | **DAT-Timing** | **Reference standard** | **Target Condition** | **Reason for exclusion from metanalysis** |
| 1 | Ilonze 202(22)  Retrospective United kingdom | 78 | 37.8 (2.4) | All blood group ^c^ | Cord | Bilirubin | Phototherapy | Data on DAT negative - NS;  39% of infants with DAT positive required phototherapy |
| 2 | Rahmati 2022(23)  Retrospective  Iran | 352 | Late-Preterm and Term | All blood group ^c^ | Cord | Bilirubin | Phototherapy,  DVET | Non-ABO / Rh incompatibility;  Phototherapy: sensitivity - 76.7%, specificity - 84.8%  DVET: sensitivity - 82.5%, specificity - 58.0% |
| 3 | Mandal 2023(24)  Retrospective  India | 16 | NS | All blood group including minor ^c^ | NS | Bilirubin | Phototherapy, Blood transfusion, DVET | Irregular erythrocyte antibodies, fetal intervention, preterm included;  Phototherapy: sensitivity - 66.6%, specificity - 71.4% |
| 4 | Papacostas 2022(25)  Retrospective  USA | 772 | > 35 wks | ABO ^a^ | Cord | TcB/ Bilirubin | Need for Phototherapy within 24 hours | Data on DAT negative - NS;  26.6% of infants with DAT positive required phototherapy |
| 5 | Talwar 2022(26)  Prospective  India | 176 | Preterm and Term | ABO ^a^ | Cord | Bilirubin | Phototherapy, DVET | Preterm infants were included;  Phototherapy: sensitivity - 93%, specificity - 79.5% |
| 6 | Das 2021(27)  Prospective India | 885 | NS | All blood group ^c^ | Cord | Bilirubin | Phototherapy | Non-ABO/Rh incompatibility;  Phototherapy: sensitivity - 20.6%, specificity - 90.9% |
| 7 | Varal 2020(28)  Retrospective  Turkey | 221 | 32 - 37 wks | All blood group ^c^ | Cord | Bilirubin | Phototherapy | Non-ABO/Rh incompatibility;  Phototherapy: sensitivity - 14.7%, specificity - 97.8% |
| 8 | Okur 2019(29)  Retrospective  Turkey | 149 | 38.2 (2.1) wks | All blood group ^c^ | Cord | Bilirubin | DVET, IVIG | Non-ABO/Rh incompatibility;  DVET: sensitivity - 80%, specificity -54.0% |
| 9 | Christensen 2018(30)  Retrospective  USA | 1174 | NS | ABO ^a^ | NS | Bilirubin | Readmission for Phototherapy | Need for phototherapy - NS;  Readmission for phototherapy: sensitivity - 10.2%. |
| 10 | Aktas 2018(31)  Prospective  Turkey | 418 | 38.6 wks | ABO and Rh ^b^ | NS | Bilirubin | Phototherapy | Data on DAT negative - NS;  4/5 (80%) DAT positive infants required phototherapy |
| 11 | Jones 2017(32)  Retrospective  United Kingdom | 1411 | 36 – 44 wks | ABO and Rh ^b^ | Cord | Bilirubin | Threshold above Phototherapy range (NICE) | Need for phototherapy - NS;  Clinical significant NNH: sensitivity - 27% |
| 12 | Karagol 2012(33)  Retrospective  Turkey | 106 | 37- 42 wks | Minor blood group | NS | Bilirubin | Phototherapy, DVET, IVIG | Only minor blood group;  DVET: sensitivity - 57.8%, specificity - 78.1% |
| 13 | Kaplan 2010(34)  Prospective  Israel | 164 | > 37 wks | ABO ^a^ | Cord | Bilirubin | Phototherapy | Data on DAT negative- NS;  49% infants with DAT positive required phototherapy |
| 14 | Watchko 2009(35)  Prospective  United States | 452 | 39 (37 - 40) wks | All blood group ^c^ | Cord | Bilirubin | DVET | Non-ABO / Rh incompatibility;  DVET: sensitivity - 100%, specificity - 44.2% |
| 15 | Dinesh 2005(36)  Retrospective  New Zealand | 94 | NS | All blood group ^c^ | Cord | Bilirubin | Phototherapy | Non-ABO/Rh incompatibility;  Preterm infants were included;  Phototherapy: sensitivity - 15.8%, specificity - 98.5% |
| 16 | Cianciarullo 2003(37)  Retrospective  Brazil | 1692 | NS | All blood group ^c^ | Cord | Bilirubin | Phototherapy, DVET | Non-ABO/Rh incompatibility;  Phototherapy: sensitivity - 60%, specificity - 58.2%  DVET: sensitivity - 93.3%, specificity - 52.0% |
| 17 | Herschel 2002(38)  Prospective  USA | 660 | 38.9 wks | All blood group ^c^ | Cord | Bilirubin | Significant Jaundice (>75th percentile) | Need for phototherapy - NS;  Significant jaundice: sensitivity - 53%, specificity - 89%. |
| 18 | Chen 1994(39)  Retrospective  China | 88 | Term | All blood group ^c^ | Cord | Bilirubin | DVET | Non-ABO/Rh incompatibility  DVET: sensitivity - 100%, specificity - 84.9% |
| 19 | Ozolek 1994(40)  Prospective  USA | 343 | NS | All blood group ^c^ | Cord | Bilirubin | Peak bilirubin >12.8 mg/dl | Need for phototherapy - NS ;  Bilirubin > 12.8mg/dl: sensitivity - 55%, specificity - 71%. |
| 20 | Procianoy 1987(41)  Brazil | 132 | 39.4 wks | ABO ^a^ | Cord | Bilirubin | Anaemia and Bilirubin > 15mg/dl | Need for phototherapy - NS ;  Anemia and bilirubin > 15mg/dl: sensitivity - 64.7%, specificity - 74.5% |
| 21 | Levine 1985(42)  Prospective  USA | 135 | NS | ABO ^a^ | Cord | Bilirubin | Peak bilirubin >12mg/dl | Need for phototherapy -NS;  Bilirubin > 12mg/dl: sensitivity - 48%, specificity - 51% |
| 22 | Dufour 1979(43)  Retrospective  United States | 246 | NS | All blood group ^c^ | Cord | Bilirubin | Phototherapy | Non-ABO/Rh incompatibility;  Phototherapy: sensitivity - 94.7%, specificity - 18.5% |
| 23 | Risemberg 1977(44)  Prospective  United States | 91 | Term | All blood group ^c^ | Cord | Bilirubin | DVET | Non-ABO/Rh incompatibility;  DVET: sensitivity - 92.3%, specificity - 82.4% |

**Abbreviations**: wks: weeks, DAT: Direct antiglobulin test, TcB : transcutaneous bilirubin, NS: not specified, IVIG: Intravenous immunoglobulin, DVET: double volume exchange transfusion, NICE: National Institute for Health and Care Excellence, Rh: Rhesus

a. ABO incompatibility: Mother blood group- O; Rh positive and neonate blood group - A or B; Rh positive or negative

b. ABO / Rh incompatibility: Mother blood group- O; Rh positive and neonate blood group- A or B ; Rh positive or negative OR Mother blood group- A, B, AB, O ; Rh negative and neonate blood group- A, B, AB, O ; Rh positive

c. All blood group: Mother blood group- A, B, AB, O ; Rh positive or negative and neonate blood group- A, B, AB, O ; Rh positive or negative

**References**

1. Lieberman L, Callum J, Cohen R, Cserti-Gazdewich C, Ladhani NNN, Buckstein J, et al. Impact of red blood cell alloimmunization on fetal and neonatal outcomes: A single center cohort study. Transfusion. 2020;60(11):2537-46.

2. Bhutani VK, Stark AR, Lazzeroni LC, Poland R, Gourley GR, Kazmierczak S, et al. Predischarge screening for severe neonatal hyperbilirubinemia identifies infants who need phototherapy. J Pediatr. 2013;162(3):477-82.e1.

3. Dillon A, Chaudhari T, Crispin P, Shadbolt B, Kent A. Has anti-D prophylaxis increased the rate of positive direct antiglobulin test results and can the direct antiglobulin test predict need for phototherapy in Rh/ABO incompatibility? J Paediatr Child Health. 2011;47(1-2):40-3.

4. Lehlimi M, El Korchi Z, Chemsi M, Badre A, Habzi A, Benomar S. L’incompatibilité fœto-maternelle dans le système ABO. Journal de Pédiatrie et de Puériculture. 2020;33(3):151-7.

5. Köse Çetinkaya A, Kahvecioğlu D. Nucleated red blood cell levels in the diagnosis of patients with indirect hyperbilirubinemia. Minerva pediatrics. 2021.

6. R Pocock EJ, K Madgwick. Maternal red cell antibodies: incidence and outcomes from a single-centre in North London. British Journal of Haematology 2017;176:5-145.

7. Peeters B, Geerts I, Van Mullem M, Micalessi I, Saegeman V, Moerman J. Post-test probability for neonatal hyperbilirubinemia based on umbilical cord blood bilirubin, direct antiglobulin test, and ABO compatibility results. European Journal of Pediatrics. 2016;175(5):651-7.

8. Kukreja GR, L.; Ahmed, Z.; Ozgonenel, B. Significance of the positive direct antibody test in neonatal ABO incompatibility. Pediatric Blood and Cancer 2014;61(0).

9. Rao LB, Ahmed Z, Ozgonenel B. The Clinical Spectrum of ABO Incompatibility and Hemolytic Disease in the Newborn. Blood. 2012;120(21):1182.

10. Larson RaA, C. A. Utility of cord blood testing in O positive mothers. Transfusion. 2017;57:128A.

11. Howard H, Martlew V, McFadyen I, Clarke C, Duguid J, Bromilow I, et al. Consequences for fetus and neonate of maternal red cell allo-immunisation. Arch Dis Child Fetal Neonatal Ed. 1998;78(1):F62-6.

12. Vilambil S, Dharmadas M, Usha K, Panthiyil Shahulhameed S, James C, Sasikala A, et al. IMMUNOHAEMATOLOGICAL PROFILE OF MATERNAL ANTIBODY-MEDIATED ABO HAEMOLYTIC DISEASE OF FOETUS AND NEWBORN. Journal of Evolution of Medical and Dental Sciences. 2017;6:5107-12.

13. Elsaie AL, Taleb M, Nicosia A, Zangaladze A, Pease ME, Newton K, et al. Comparison of end-tidal carbon monoxide measurements with direct antiglobulin tests in the management of neonatal hyperbilirubinemia. J Perinatol. 2020;40(10):1513-7.

14. Zhang F, Chen L, Shang S, Jiang K. A clinical prediction rule for acute bilirubin encephalopathy in neonates with extreme hyperbilirubinemia: A retrospective cohort study. Medicine (Baltimore). 2020;99(9):e19364.

15. Tıraş M, Can E, Hamilçıkan Ş. Role of Cord Blood Carboxyhemoglobin in Detecting Significant Hyperbilirubinemia in Term Neonates with ABO Alloimmunization. Am J Perinatol. 2022;39(12):1321-5.

16. Bel Hadj I, Boukhris MR, Khalsi F, Namouchi M, Bougmiza I, Tinsa F, et al. ABO hemolytic disease of newborn : Does newborn's blood group a risk factor. La Tunisie médicale. 2019;97:455-60.

17. Margolis I, DeRuyte D. 131 Direct Antiglobulin Titer (DAT) Strength and Hyperbilirubinemia in Canadian Infants. Paediatr Child Health. 24: © The Author(s) 2019. Published by Oxford University Press on behalf of the Canadian Paediatric Society. All rights reserved. For permissions, please e-mail: journals.permissions@oup.com.; 2019. p. e51-2.

18. Schutzman DL, Gatien E, Ajayi S, Wong RJ. Carboxyhemoglobin levels as a predictor of risk for significant hyperbilirubinemia in African-American DAT(+) infants. J Perinatol. 2016;36(5):386-8.

19. Oztekin O, Kalay S, Tezel G, Barsal E, Bozkurt S, Akcakus M, et al. Is the strength of direct antiglobulin test important for the duration of phototherapy? The journal of maternal-fetal & neonatal medicine : the official journal of the European Association of Perinatal Medicine, the Federation of Asia and Oceania Perinatal Societies, the International Society of Perinatal Obstet. 2014;27(5):534-6.

20. Arevalo C, Herrmann F, Aguirre E, Vera J. [Hemolytic disease of the newborn due to ABO incompatibility. A predictive test]. Enfermedad hemolitica del recien nacido por incompatibilidad ABO Prueba predictiva. 1992;120(2):163-7.

21. Kirkman HN, Jr. Further evidence for a racial difference in frequency of ABO hemolytic disease. J Pediatr. 1977;90(5):717-21.

22. Ilonze J, Kannan Loganathan P, Kumar R, Elliot C. Does strengths of a positive direct antiglobulin test predicts the need for phototherapy and duration of phototherapy?–a single center, retrospective study. Journal of Maternal-Fetal and Neonatal Medicine. 2023;36(2).

23. Rahmati A, Farhat AS, Boroumand-Noughabi S, Soleymani F, Keramati M. Retrospective analysis of direct antiglobulin test positivity at tertiary academic hospital over 10 years. Transfus Apher Sci. 2022;61(3):103358.

24. Mandal S, Kaur D, Negi G, Basu S, Chaturvedi J, Maji M, et al. Irregular erythrocyte antibodies among antenatal women and their neonatal outcome at a tertiary care hospital in Northern India. Postgrad Med J. 2023;99(1169):145-52.

25. Papacostas MF, Robertson DM, McLean MD, Wolfe KD, Liu H, Shope TR. Sixth-Hour Trancutaneous Bilirubin and Need for Phototherapy in DAT Positive Newborns. Pediatrics. 2022;149(3):e2021054071.

26. Talwar M, Jain A, Sharma RR, Kumar P, Saha SC, Singh L. The spectrum of ABO haemolytic disease of the fetus and newborn in neonates born to group O mothers. Vox Sang. 2022;117(9):1112-20.

27. Das S, Shastry S, Chakravarthy PK, Baliga PB. Clinical Implication of Immunohaematological Tests in ABO haemolytic disease of newborn: Revisiting an old disease. Transfusion Medicine. 2021;31(1):30-5.

28. Guney Varal I, Mengi S, Dogan P, Tutanc M, Bostanci M, Cizmeci MN. Elevated blood carboxyhemoglobin levels as an early predictor of phototherapy requirement in moderate and late preterm infants. Journal of Maternal-Fetal and Neonatal Medicine. 2020;33(8):1441-6.

29. Okur N, Büyüktiryaki M, Uras N, Öncel MY, Ertekin Ö, Canpolat FE, et al. Effectiveness of light emitting diode phototherapy for direct coombs positive newborns. Journal of Harran University Medical Faculty. 2019;16(2):169-73.

30. Christensen RD, Baer VL, MacQueen BC, O'Brien EA, Ilstrup SJ. ABO hemolytic disease of the fetus and newborn: thirteen years of data after implementing a universal bilirubin screening and management program. J Perinatol. 2018;38(5):517-25.

31. Aktas S, Dogan C, Okmen ZH, Gulec SG. Is Cord Blood Bilirubin Level a Reliable Predictor for Developing Significant Hyperbilirubinemia? Am J Perinatol. 2019;36(3):317-21.

32. Jones KDJ, Grossman SE, Kumaranayakam D, Rao A, Fegan G, Aladangady N. Umbilical cord bilirubin as a predictor of neonatal jaundice: a retrospective cohort study. BMC Pediatr. 2017;17(1):186.

33. Karagol BS, Zenciroglu A, Okumus N, Karadag N, Dursun A, Hakan N. Hemolytic disease of the newborn caused by irregular blood subgroup (Kell, C, c, E, and e) incompatibilities: Report of 106 cases at a tertiary-care centre. American Journal of Perinatology. 2012;29(6):449-54.

34. Kaplan M, Hammerman C, Vreman HJ, Wong RJ, Stevenson DK. Hemolysis and hyperbilirubinemia in antiglobulin positive, direct ABO blood group heterospecific neonates. J Pediatr. 2010;157(5):772-7.

35. Watchko JF, Lin Z, Clark RH, Kelleher AS, Walker MW, Spitzer AR. Complex multifactorial nature of significant hyperbilirubinemia in neonates. Pediatrics. 2009;124(5):e868-77.

36. Dinesh D. Review of positive direct antiglobulin tests found on cord blood sampling. J Paediatr Child Health. 2005;41(9-10):504-7.

37. Cianciarullo MA, Ceccon ME, Vaz FA. Prevalence of immunohematologic tests at birth and the incidence of hemolytic disease in the newborn. Revista da Associacao Medica Brasileira (1992). 2003;49(1):45-53.

38. Herschel M, Karrison T, Wen M, Caldarelli L, Baron B. Evaluation of the direct antiglobulin (Coombs') test for identifying newborns at risk for hemolysis as determined by end-tidal carbon monoxide concentration (ETCOc); and comparison of the Coombs' test with ETCOc for detecting significant jaundice. J Perinatol. 2002;22(5):341-7.

39. Chen JY, Ling UP. Prediction of the development of neonatal hyperbilirubinemia in ABO incompatibility. Zhonghua yi xue za zhi = Chinese medical journal; Free China ed. 1994;53(1):13-8.

40. Ozolek JA, Watchko JF, Mimouni F. Prevalence and lack of clinical significance of blood group incompatibility in mothers with blood type A or B. J Pediatr. 1994;125(1):87-91.

41. Procianoy RS, Giacomini CB, Farina DM, Mollin GA, Winckler MI, Silveira MB, et al. Early diagnosis of ABO haemolytic disease of the newborn. Eur J Pediatr. 1987;146(4):390-3.

42. Levine DH, Meyer HB. Newborn screening for ABO hemolytic disease. Clin Pediatr (Phila). 1985;24(7):391-4.

43. Dufour DR, Monoghan WP. ABO hemolytic disease of the newborn. A retrospective analysis of 254 cases. American journal of clinical pathology. 1980;73(3):369-73.

44. Risemberg HM, Mazzi E, MacDonald MG, Peralta M, Heldrich F. Correlation of cord bilirubin levels with hyperbilirubinaemia in ABO incompatibility. Archives of disease in childhood. 1977;52(3):219-22.
